# Lysine acetyltransferase 8-mediated histone acetylation, regulated by GBA1, is associated with lysosomal function related to α-Synuclein pathology

**DOI:** 10.1038/s41419-026-08928-2

**Published:** 2026-06-08

**Authors:** Yifan Cao, Zhixiong Zhang, Xinbei Gu, Haifeng Lu, Jin Wu, Chuang Zhang, Xinyue Huang, Haitao Qin, Feng Liu, Zhenhua Liu, Beisha Tang, Xiaojun Lu, Haigang Ren, Hongyang Sun, Rui Wang, Guanghui Wang

**Affiliations:** 1 Laboratory of Molecular Neuropathology, Department of Pharmacology, Jiangsu Key Laboratory of Drug Discovery and Translational Research for Brain Diseases, College of Pharmaceutical Sciences, Soochow University, Suzhou, Jiangsu China; 2 Department of Neurology, the First Affiliated Hospital of Soochow University, Suzhou, Jiangsu China; 3 Jiangsu Key Laboratory of Translational Research and Therapy for Neuro-Psycho-Diseases and Department of Medicinal Chemistry, College of Pharmaceutical Sciences, Soochow University, Suzhou, Jiangsu China; 4 Department of Neurology & National Clinical Research Centre for Geriatric Disorders, Xiangya Hospital, Central South University, Changsha, Hunan China; 5 Suzhou Key Laboratory of Geriatric Neurological Disorders, Department of Neurosurgery, the First People’s Hospital of Taicang, Taicang Affiliated Hospital of Soochow University, Suzhou, Jiangsu China; 6 MOE Key Laboratory of Geriatric Diseases and Immunology, Soochow University, Suzhou, Jiangsu China; 7 Suzhou Key Laboratory of Geriatric Neurological Disorders, Center of Translational Medicine, the First People’s Hospital of Taicang, Taicang Affiliated Hospital of Soochow University, Suzhou, Jiangsu China

**Keywords:** Cell death in the nervous system, Parkinson's disease

## Abstract

Lysosomal defects are closely linked to Parkinson’s disease (PD). Mutations in the *GBA1* gene, encoding the lysosomal enzyme glucocerebrosidase (GCase), are major genetic risk factors for PD. *GBA1* deficiency causes lysosomal dysfunction, leading to α-synuclein (α-syn) accumulation and PD progression. However, the underlying mechanisms remain unclear. In this study, we identified a novel GBA1-KAT8 regulatory pathway that controls lysosomal activity. GBA1 overexpression enhances lysosomal enzyme expression, regulates histone H4 acetylation at K16 via KAT8, and promotes lysosome-associated gene expression, highlighting an epigenetic mechanism in lysosomal biogenesis. Furthermore, GBA1 upregulated KAT8 expression, increased lysosomal enzyme levels, and decreased PFF-induced α-syn accumulation both in vitro and in vivo. The involvement of KAT8 as a critical acetyltransferase that modulates nuclear–lysosomal signaling pathways provides a mechanistic explanation for *GBA1* deficiency-induced lysosomal dysfunction in association with PD pathology.

## Introduction

Parkinson’s disease (PD) is a neurodegenerative disorder characterized by motor symptoms such as rigidity, tremor, bradykinesia, and postural instability, as well as nonmotor symptoms such as cognitive impairments, anxiety, depression, and dementia [1]. With disease progression, there is a progressive loss of dopaminergic neurons in the substantia nigra pars compacta, accompanied by the accumulation of α-synuclein (α-syn) in Lewy bodies and Lewy neurites across various brain regions [2]. Lysosomes, which are membrane-bound organelles with an acidic lumen containing hydrolytic enzymes, are primarily responsible for degrading macromolecules and clearing damaged organelles. Dysfunction of lysosomal proteolytic systems is believed to contribute to the accumulation of pathological α-syn aggregation in PD [3–5].

Genetic insights from genome-wide association studies (GWASs) have strengthened the connection between lysosomal abnormalities and PD susceptibility, identifying multiple risk loci encoding lysosomal components [6–8]. Among the identified risk loci, genes such as *ATP13A2* and *TMEM175*, which are essential for maintaining lysosomal pH homeostasis, are significantly associated with PD pathogenesis [9, 10]. The lysosomal enzymes *CTSB* (cathepsin B) and *CTSD* (cathepsin D) are important for lysosomal proteolysis, which are tightly associated with α-syn pathology [5, 6, 11, 12]. A study utilizing correlative light and electron microscopy (CLEM) for the morphological analysis of Lewy bodies in PD patients provided more direct evidence linking lysosomal dysfunction with α-syn pathology, showing that Lewy bodies are composed of large amounts of α-synuclein, lipids, and lysosomal structures [13]. In addition, in lysosomal storage disorders (LSDs), in which the deficiencies of lysosomal enzymes lead to lysosomal dysfunction, α-syn pathology is present in the brains of patients with various LSDs, further suggesting that there is a close link between lysosomal dysfunction and PD pathology [14–17].

Among genetic risk factors, mutations in the *GBA1* gene, which are linked to the lysosomal storage disorder Gaucher’s disease, are recognized as the most common genetic risk factor for PD [18, 19]. The *GBA1* gene encodes glucocerebrosidase (GCase), a lysosomal enzyme responsible for degrading glucosylceramide and glucosylsphingosine [20]. Deficiency of GCase leads to the accumulation of the lipid substrates glucosylceramide and glucosylsphingosine, which are associated with the aggregation of α-syn [21–23]. Furthermore, α-syn accumulation may disturb the trafficking of newly synthesized GCase from the endoplasmic reticulum into lysosomes, contributing to a positive pathogenic feedback loop, which further induces α-syn accumulation [24]. Despite conclusive evidence positioning lysosomal failure as a central driver of PD pathophysiology [25, 26] and preclinical validation of GCase activation strategies for α-syn clearance [27, 28], the precise cascade through which GCase insufficiency compromises lysosomal fidelity remains enigmatic.

In this study, we demonstrated that GBA1 upregulates KAT8 to induce lysosomal biogenesis, which represses α-syn PFF-induced α-syn pathology and neurodegeneration in vitro and in vivo. Thus, our study elucidates the mechanistic connections among GCase activity, lysosomal homeostasis, and α-syn pathology.

## Results

### α-Syn PFFs cause lysosomal dysfunction

**Fig. 1:**
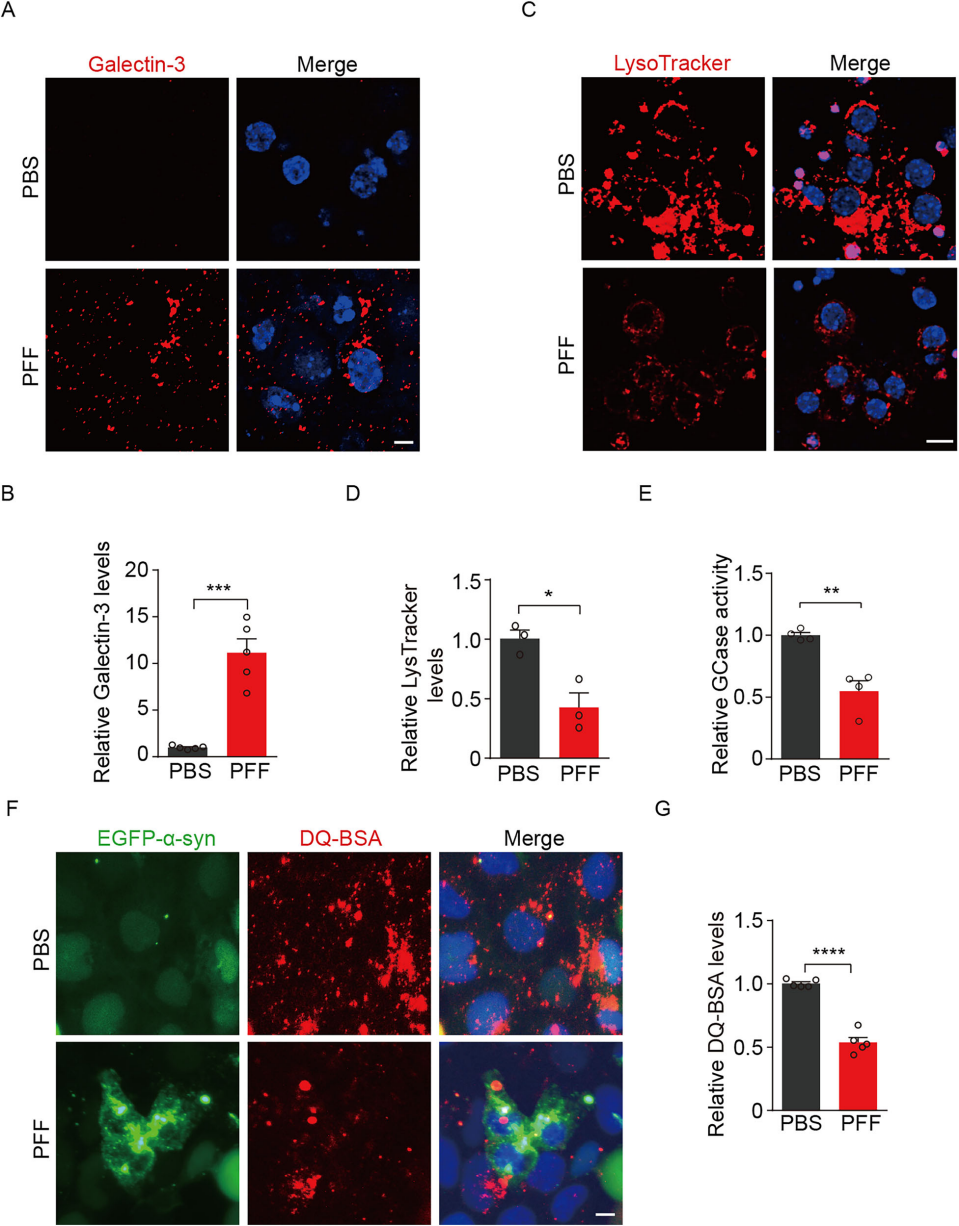
α-Syn PFFs cause lysosomal dysfunction. **A**–**D** Primary neurons at 7 DIV were treated with PBS or 1 µg/mL PFFs for 14 days and then processed for analysis. Representative images of immunofluorescence staining for galectin-3 and DAPI in (**A**). Scale bar, 20 μm. Quantification of the fluorescence intensity of galectin-3 in (**B**). (***p < 0.001, n = 5/group). Representative images of LysoTracker staining in (**C**). Scale bars, 20 μm. Quantification of the LysoTracker intensity in (**D**). (*p < 0.05, *n* = 3/group). **E** Quantification of the GCase activity levels. (**p < 0.01, n = 4/group). **F** EGFP-α-syn-HEK293 cells were treated with 1 µg/mL PFFs for 24 h, and DQ-BSA was incubated with the cells at a final concentration of 50 µg/mL in prewarmed medium for 8 h. Representative images of DQ-BSA staining. Scale bar, 10 μm. **G** Quantification of the DQ-BSA intensity. (****p < 0.0001, *n* = 5/group). The data are presented as the means ± SEMs. Statistical significance was determined by an unpaired *t* test in (**B**–**E**) and (**G**).

To investigate the effects of α-syn PFFs on lysosomal function, we treated primary cortical neurons derived from wild-type (WT) C57BL6 mice with α-syn PFFs, after which the neurons were cultured for 7 days in vitro (DIV) and then exposed to α-syn PFFs for 14 days. Lysosomal damage was assessed by staining for galectin-3, a marker of endolysosomal membrane damage, which revealed increased galectin-3 puncta formation following α-syn PFF treatment (Fig. 1A, B). Disruption of the lysosomal membrane leads to a pH imbalance. LysoTrack Red, a lysosensor that indicates a lysosomal acidic environment, significantly decreased the lysosensor fluorescence intensity in α-syn PFF-treated neurons compared with that in PBS-treated control neurons (Fig. 1C, D), suggesting an increase in lysosomal pH after α-syn PFF treatment. Additionally, we observed a decrease in GCase activity following α-syn PFF treatment (Fig. 1E), further suggesting an impairment of lysosomal function. To further assess the changes in lysosomal proteolytic activity, we constructed a cellular model using human embryonic kidney (HEK293) cells that stably overexpress EGFP-tagged human wild-type α-synuclein (EGFP-α-syn-HEK293 cells). After treatment with α-syn PFFs, the aggregation of EGFP-α-syn can be visualized [29]. In α-syn PFF-treated cells, EGFP-α-syn aggregates were observed (Fig. 1F). Moreover, when DQ-BSA-Red, a red fluorescent dye that emits bright fluorescence when it is cleaved by lysosomal proteases, was used, we observed a significant decrease in DQ-BSA fluorescence intensity in α-syn PFF-treated cells compared with untreated controls, indicating defects in lysosomal proteolysis (Fig. 1F, G).

### GBA1 restores lysosomal function by promoting lysosomal biogenesis

**Fig. 2:**
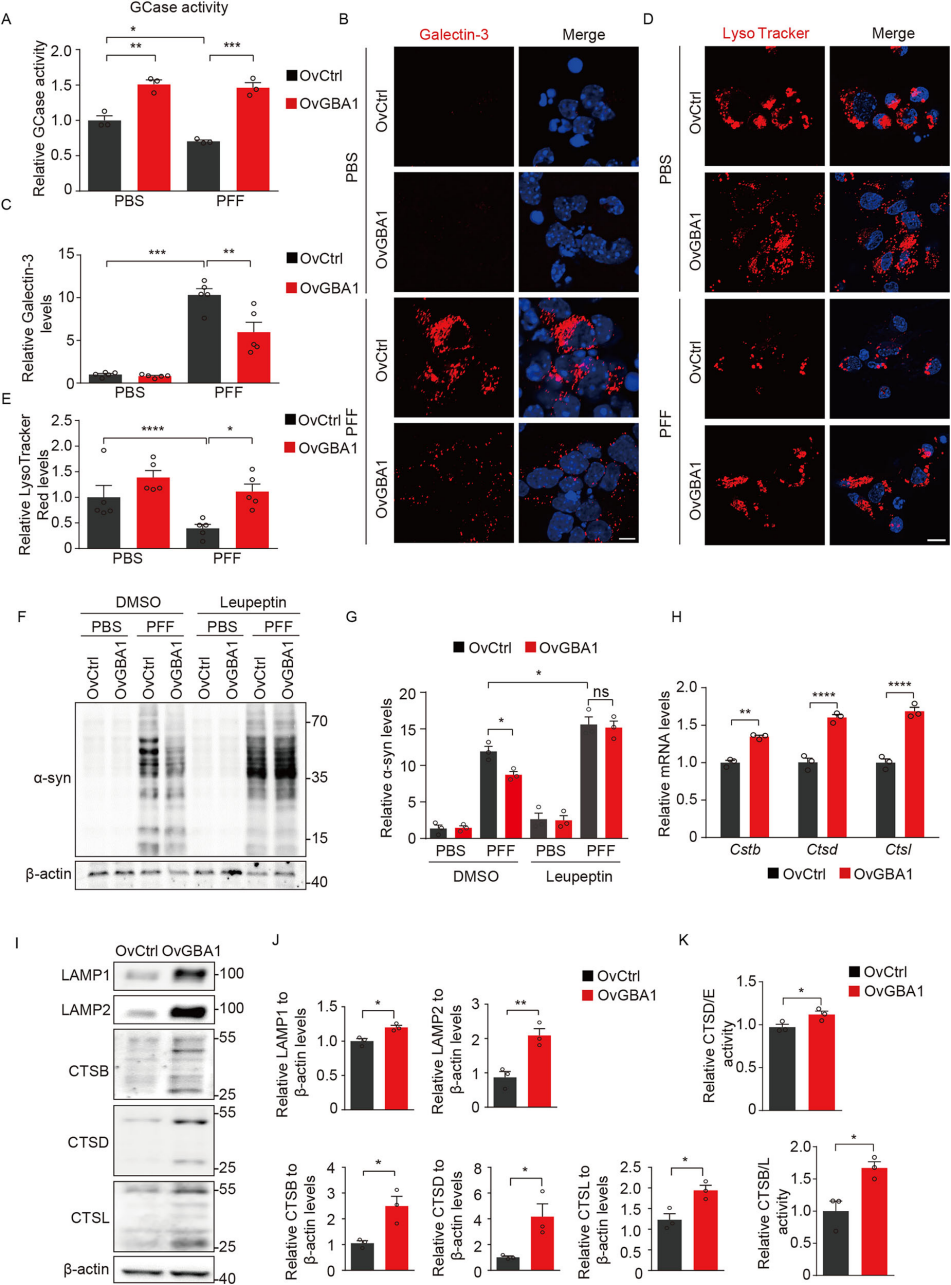
Overexpression of GBA1 rescues lysosomal dysfunction caused by α-syn PFFs. **A**–**E** Primary neurons at 6 DIV were transduced with LV containing *Flag*-*GBA1* or *Flag*. PBS or 1 µg/mL PFFs were added to the neurons at 7 DIV for 14 days, after which they were processed for analysis. Quantification of GCase activity in (**A**). (*p < 0.05, *n* = 4/group). Representative images of immunofluorescence staining with galectin-3 in (**B**). Scale bar, 10 μm. Quantification of the fluorescence intensity of galectin-3 in (**C**). (**p < 0.01, ***p < 0.001, *n* = 5/group). Representative images of LysoTracker staining in (**D**). Scale bar, 10 μm. Quantification of the LysoTracker intensity in (**E**). (*p < 0.05, ****p < 0.0001, n = 5/group). **F,** **G** Primary neurons were pretreated with leupeptin (1 μM) at 5 DIV and then transduced with LVs containing *EGFP*-*GBA1* or *EGFP* at 6 DIV. PBS or 1 µg/mL PFFs were added to the neurons at 7 DIV for 14 days. After treatment, the neuron lysates were sequentially extracted with 1% TX-100 followed by 2% SDS (TX-insoluble). Immunoblots of neuron lysates containing misfolded α-syn. β-actin served as a loading control in (**F**). Quantification of misfolded α-syn protein levels normalized to that of β-actin in (**G**). (*p < 0.05, ns indicates not significant, *n* = 3/group). Primary neurons at DIV6 were transduced with LVs containing *EGFP*-*GBA1* or *EGFP* for 5 days and then processed for analysis. qPCR analysis of the mRNA levels of lysosomal-related genes (*Ctsb, Ctsd, and Ctsl*) in (**H**). (**p < 0.01, ***p < 0.001, ****p < 0.0001, *n* = 3/group). Lysosomal-related protein levels were visualized by immunoblotting in (**I**), and the quantified protein levels were normalized to those of β-actin in (**J**). (*p < 0.05, **p < 0.01, *n* = 3/group). Quantification of the activities of the indicated lysosomal cathepsin activities in (**K**). (*p < 0.05, *n* = 3/group). The data are presented as the means ± SEMs. Statistical significance was determined by an unpaired *t*-test in (**H**), (**J**), and (**K**) or two-way ANOVA in (**A**), (**C**), (**E**) and (**G**).

Given that α-syn PFFs lead to lysosomal damage and decreased GCase activity, we wondered whether the overexpression of *GBA1* could improve lysosomal function. We constructed lentivirus (LV) vectors encoding *GBA1* and transduced primary cortical neurons. The overexpression of *GBA1* significantly increased the activity of GCase in primary neurons and effectively restored the activity impaired by α-syn PFFs (Fig. 2A). Immunocytochemical staining for galectin-3 revealed that the overexpression of *GBA1* blocked α-syn PFF-induced lysosomal membrane damage (Fig. 2B, C). Moreover, overexpression of *GBA1* prevented the decrease in lysosomal acidity that was induced by α-syn PFFs, as indicated by LysoTracker (Fig. 2D, E). To determine whether an improvement in lysosomal function by *GBA1* overexpression has an impact on α-syn pathology, we examined α-syn pathology via an α-syn PFF-based aggregation model. The overexpression of *GBA1* repressed α-syn aggregation in Triton X-100 (TX-100)-insoluble and SDS-soluble fractions (Fig. 2F, G). Moreover, leupeptin, a protease inhibitor that blocks lysosomal function, blocked the repressive effects of *GBA1* overexpression on PFF-induced α-syn aggregation, suggesting that lysosomal protease activity is involved in *GBA1*-induced α-syn pathology (Fig. 2F, G). Immunocytochemical staining also revealed that *GBA1* overexpression mitigated the PFF-induced pathology of α-syn, which was labeled with an antibody against S129-phosphorylated α-syn (Fig. S1A, B). Similar results were obtained via Western blot analysis (Fig. S1C–F). We further validated the neuroprotective effect of *GBA1* overexpression on α-syn PFFs. Lactate dehydrogenase (LDH) release assays revealed that *GBA1* overexpression inhibited PFF-induced neurotoxicity (Fig. S1G). Immunocytochemical staining with an antibody against NeuN, a specific neuronal marker, revealed that *GBA1* overexpression inhibited PFF-induced neuronal loss (Fig. S1H). Lysosomal cathepsins are critical for α-syn degradation [11, 30, 31]. We therefore examined the expression of cathepsins in cells in which *GBA1* was overexpressed. In primary neurons, the overexpression of *GBA1* led to increases in both the mRNA and protein levels of lysosomal cathepsins (Fig. 2H–J). Moreover, the activities of cathepsins and lysosomal proteases also increased (Figs. 2K, S1I, J). Moreover, the expression of the lysosome-associated proteins LAMP1 and LAMP2 was also significantly upregulated (Fig. 2I, J), suggesting an overall increase in lysosomal biogenesis after *GBA1* overexpression.

### GBA1 increases lysosomal function through the histone acetyltransferase KAT8

**Fig. 3:**
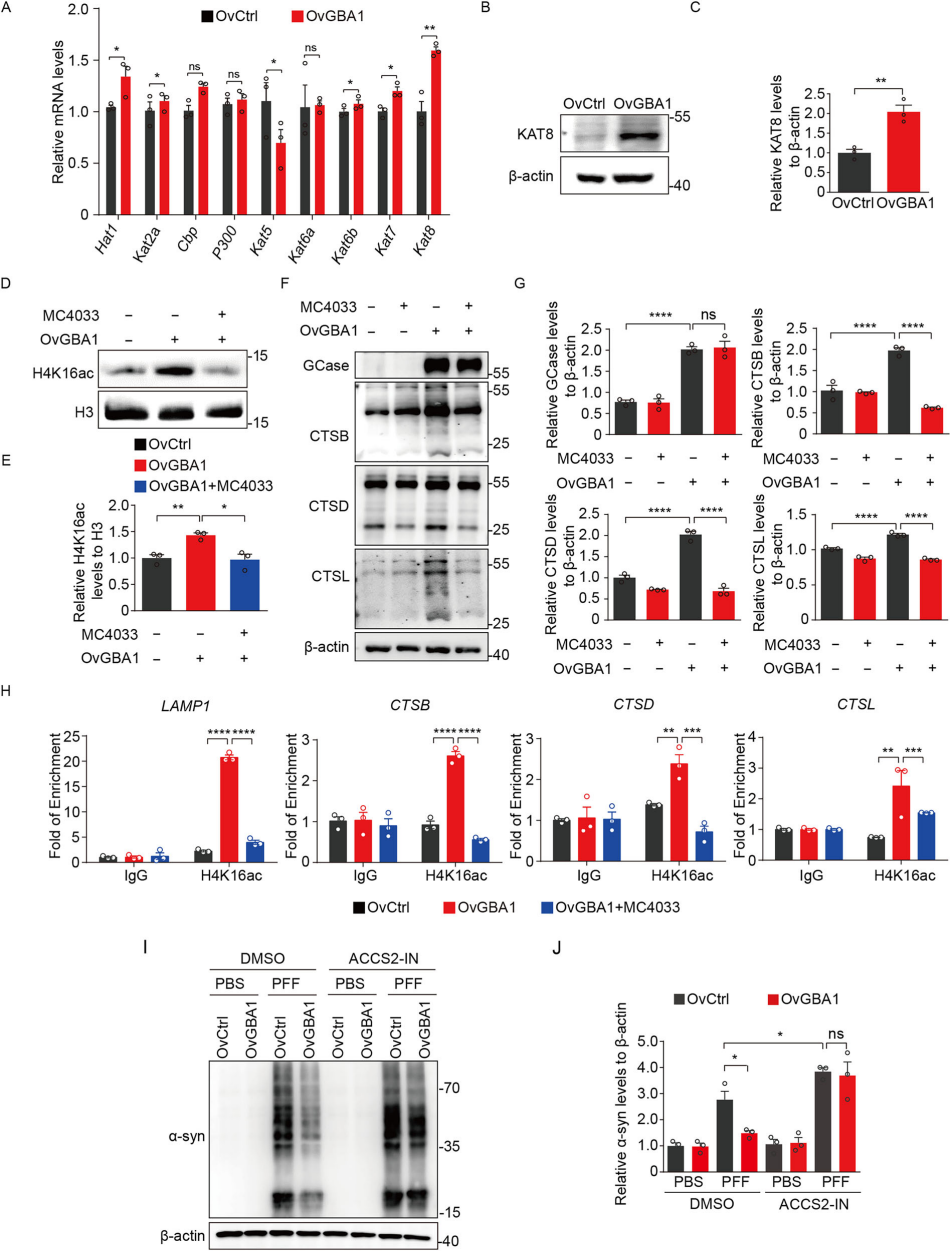
GBA1 overexpression increases lysosomal function through the histone acetyltransferase KAT8. **A**–**C** Primary neurons at DIV6 were transduced with LVs containing *EGFP*-*GBA1* or *EGFP* for 5 days and then processed for analysis. qPCR analysis of the mRNA levels of the KAT family in (**A**). (*p < 0.05, **p < 0.01, ns indicates not significant, *n* = 3/group). KAT8 protein levels were visualized by immunoblotting in (**B**), and KAT8 protein levels were normalized to those of β-actin in (**C**). (**p < 0.01, *n* = 3/group). **D**–**G** MC4033 (20 μM) was used to pretreat primary neurons at DIV5, which were then transduced with LVs containing *EGFP*-*GBA1* or *EGFP* for 5 days. The H4K16ac level was visualized by immunoblotting in (**D**), and the H4K16ac levels were normalized to the H3 level in (**E**). (*p < 0.05, **p < 0.01, *n* = 3/group). The lysosomal-related protein levels were visualized by immunoblotting in (**F**), and the quantified protein levels were normalized to those of β-actin in (**G**). (****p < 0.0001, ns indicates not significant, *n* = 3/group). **H** ChIP‒qPCR data comparing the enrichment of H4K16ac at *CTSB*, *CTSD*, and *CTSL* in U2OS cells transfected with vectors containing *Flag*-*GBA1* or *Flag* for 3 days. IgG was used as the negative control. (**p < 0.01, ***p < 0.001, ****p < 0.0001, *n* = 3/group). **I**, **J** Primary neurons at 5 DIV were pretreated with ACSS2-IN (2.5 μM) and then transduced with LVs containing *EGFP*-*GBA1* or *EGFP* at 6 DIV. PBS or 1 µg/mL PFFs were added to the neurons at 7 DIV for 14 days. After treatment, the neuron lysates were sequentially extracted with 1% TX-100 followed by 2% SDS (TX-insoluble). Immunoblots of neuron lysates containing misfolded α-syn. β-actin served as a loading control in (**I**). Quantification of misfolded α-syn protein levels normalized to that of β-actin in (**J**). (*p < 0.05, ns indicates not significant, *n* = 3/group). The data are presented as the means ± SEMs. Statistical significance was determined by an unpaired *t*-test in (**A**) and (**C**) or two-way ANOVA in (**E**), (**G**) and (**J**).

Transcription factor EB (TFEB) is a key regulator of lysosomal biogenesis and functions primarily via its translocation from the cytoplasm to the nucleus [32–34]. To determine whether *GBA1* overexpression enhances lysosomal function through TFEB-mediated biosynthesis, we analyzed TFEB expression in the nucleus via Western blotting. In primary neurons, *GBA1* overexpression did not affect intranuclear TFEB expression in cells (Fig. S2A, B). Additionally, we transfected U2OS cells with Flag and Flag-*GBA1* plasmids and labeled them with endogenous TFEB via immunofluorescence. As a positive control for monitoring TFEB nuclear translocation, the mTOR inhibitor Torin 1 was used. In U2OS cells treated with Torin 1, TFEB nuclear translocation was observed (Fig. S2C, D). However, nuclear translocation of TFEB was not observed in *GBA1*-overexpressing cells (Fig. S2C, D), indicating that TFEB does not mediate the upregulation of lysosome-associated gene expression in *GBA1*-overexpressing cells. Gene expression regulation encompasses both transcription factor binding and chromatin modifications. Previous studies have highlighted the prevalence of histone acetylation in chromatin modifications, which promote active transcription. This modification is catalyzed by lysine acetyltransferases (KATs and HATs) and reversed by lysine deacetylases (HDACs and KDACs) [35, 36]. We wondered whether *GBA1* overexpression could enhance lysosome-associated gene expression through mechanisms linked to histone acetylation. We examined the expression levels of regulators of histone acetylation after *GBA1* was overexpressed in primary neurons. Quantitative polymerase chain reaction (qPCR) analysis revealed changes in several regulators (Fig. 3A). Among these genes, *Kat8* was more significantly increased than the other genes (such as *Kat5* and *Kat7*) after *GBA1* was overexpressed (Fig. 3A). Western blot analysis further confirmed an increase in KAT8 protein expression in *GBA1*-overexpressing neurons compared with control neurons (Fig. 3B, C). KAT8 mediates histone 4 acetylation at lysine 16 (H4K16ac) [37, 38]. In primary neurons in which *GBA1* was overexpressed, a significant increase in H4K16ac levels was observed in *GBA1*-overexpressing neurons (Fig. 3D, E), suggesting an increase in KAT8 activity. To further determine whether KAT8 mediates *GBA1* overexpression-regulated histone 4 acetylation, we inactivated KAT8 with MC4033, a selective KAT8 inhibitor. Inactivation of KAT8 abolished the upregulation of H4K16ac by *GBA1* overexpression (Fig. 3D, E). To determine whether KAT8 mediates GBA1-induced lysosomal biogenesis, we examined the lysosomal cathepsin expression in primary neurons overexpressing GBA1 in the presence or absence of the KAT8 inhibitor MC4033. The inactivation of KAT8 blocked the increase in lysosomal cathepsin expression caused by *GBA1* overexpression (Fig. 3F, G), indicating that KAT8 activity is required for the transcriptional induction of those lysosomal genes downstream of GBA1. Because other KAT family members were also modulated following GBA1 manipulation, we next examined whether KAT5 or KAT7 contributes to lysosomal gene expression similarly. Treatment of GBA1-overexpressing cells with the KAT5-specific inhibitor NU9056 or the KAT7 inhibitor WM-3835 partially suppressed the induction of *CTSB* and *CTSD*, whereas *CTSL* expression remained unaffected (Fig. S2G). These findings suggest that KAT5 and KAT7 also participate in GBA1-mediated regulation of lysosomal biogenesis, but with less efficacy than KAT8. To further evaluate functional specificity, we treated U2OS cells overexpressing GBA1 with KAT5, KAT7, and KAT8 inhibitors, and evaluated lysosomal enzyme activity using DQ-BSA degradation and Lysotracker-based acidification. The results showed that GBA1 overexpression enhanced the overall lysosomal hydrolytic activity and acidification.

Overall, the data support that KAT8, through H4K16 acetylation, acts as the major regulator in this pathway. To further examine whether KAT8-regulated H4K16ac is involved in the transcriptional activation of lysosomal genes, we performed chromatin immunoprecipitation coupled with quantitative PCR (ChIP-qPCR). Enrichment of H4K16ac at the promoters of *LAMP1* and the cathepsin genes *CTSB*, *CTSD*, and *CTSL* was observed in cells in which *GBA1* was overexpressed. Importantly, this increase in H4K16ac enrichment was markedly attenuated by treatment with the KAT8 inhibitor MC4033, suggesting that GBA1 overexpression-induced lysosomal gene expression is associated with enhanced H4K16 acetylation in a KAT8-dependent manner (Fig. 3H). Acetyl-CoA is a critical substrate for acetyltransferases. Acyl-CoA synthetase short-chain family member 2 (ACSS2) is a key enzyme that synthesizes acetyl-CoA from glucose and acetate, directly linking cellular metabolism to histone acetylation [39, 40]. ACSS2 also interacts with KAT8, which promotes H4K16 modification [41]. To further identify the effects of H4K16 modification on α-syn aggregation, we treated primary neurons with the ACSS2 inhibitor ACSS2-IN for 72 hours (h). Western blot analysis revealed a reduction in the nuclear level of H4K16ac, confirming that ACSS2 activity is crucial for maintaining H4K16ac acetylation, and with the inhibition of ACSS2, H4K16ac modification was decreased (Fig. S2E, F). In primary neurons in which *GBA1* was overexpressed, the inhibition of ACSS2 by ACSS2-IN abolished the repressive effects of *GBA1* on α-syn aggregation (Fig. 3I, J), further suggesting that H4K16ac modification by ACSS2 is involved in the regulation of α-syn aggregation by *GBA1*. Moreover, KAT8 inhibition significantly attenuated both lysosomal enzyme activity and acidification, whereas inhibition of KAT5 or KAT7 had minimal effects (Fig. S2H–K). To further explore whether KAT8 expression depends on GCase activity under pathological conditions, we treated mouse primary cortical neurons with the selective GCase inhibitor conduritol-β-epoxide (CBE). We found that inhibition of GCase activity significantly reduced the mRNA level of *Kat8* (Fig. S2L), indicating that endogenous GCase activity is required to maintain KAT8 expression in neurons. We next assessed whether pathogenic GBA1 mutants (N370S and L444P) with reduced enzyme activity have similar effects as wild-type GBA1 does. We transfected U2OS cells with GBA1 L444P or N370S mutants, and then examined KAT8 and lysosomal gene expressions, and lysosomal proteolytic activity (DQ-BSA). In contrast to wild-type GBA1, neither mutant increased the mRNA levels of KAT8 nor induced lysosome-associated genes (Fig. S2M). Consistently, lysosomal proteolytic activity was not enhanced in cells expressing either mutant (Fig. S2N, O). These findings indicate that the effects of GBA1 overexpression on the modulation of KAT8-lysosome depend on the activity of GCase.

### GBA1 upregulates the expression of KAT8 and lysosomal enzymes and decreases α-syn PFF-induced α-syn aggregation in primary neurons

**Fig. 4:**
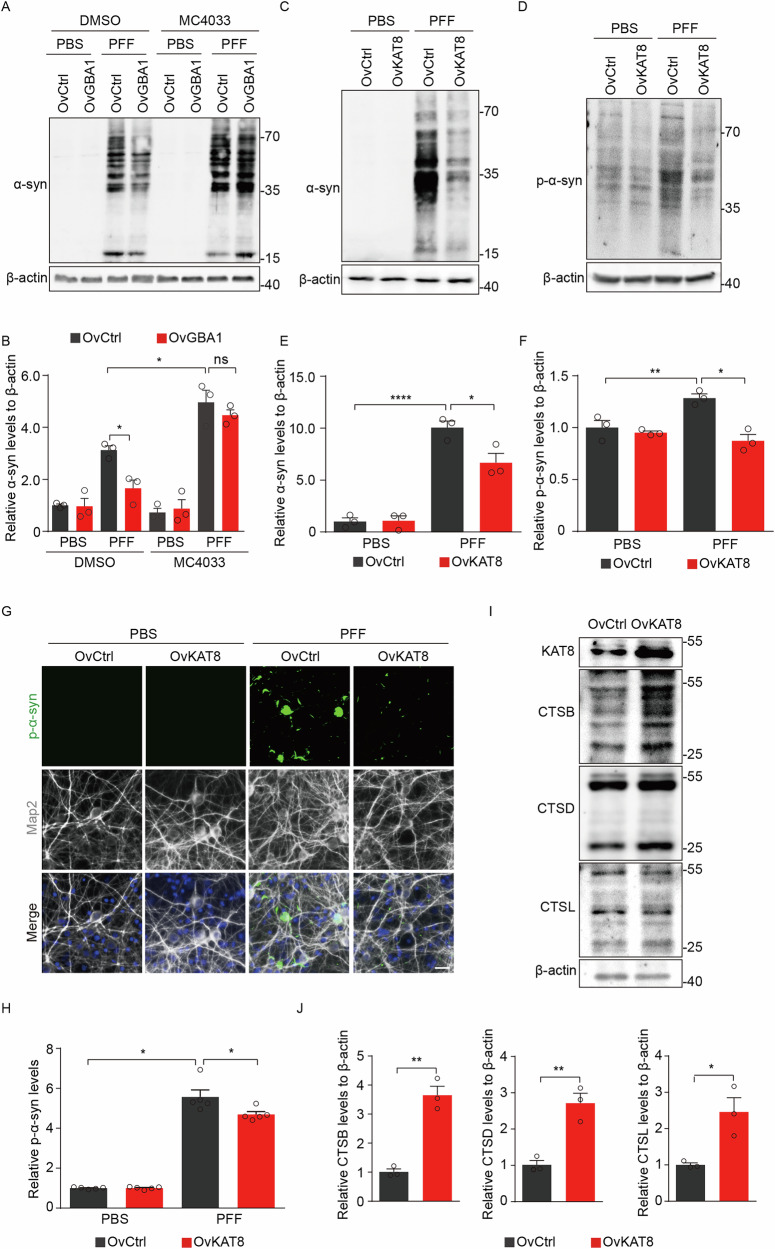
Overexpression of KAT8 rescues α-syn aggregation caused by α-syn PFF-treatment in primary neurons. **A**, **B** Primary neurons at 5 DIV were pretreated with MC4033 (20 μM) and then transduced with LVs containing *EGFP*-*GBA1* or *EGFP* at 6 DIV. PBS or 1 µg/mL PFFs were added to the neurons at 7 DIV for 14 days. After treatment, the neuron lysates were sequentially extracted with 1% TX-100 followed by 2% SDS (TX-insoluble). Immunoblots of neuron lysates containing misfolded α-syn. β-actin served as a loading control in (**A**). Quantification of misfolded α-syn protein levels normalized to that of β-actin in (**B**). (*p < 0.05, ns indicates not significant, *n* = 3/group). Primary neurons at DIV6 were transduced with LV containing *Flag*-*KAT8* or *Flag*. PBS or 1 µg/mL PFFs were added to the neurons at 7 DIV for 14 days. After treatment, the neuron lysates were sequentially extracted with 1% TX-100 followed by 2% SDS (TX-insoluble). Immunoblots of neuron lysates containing misfolded α-syn (**C**) and p-α-syn (**E**) are shown. β-actin served as a loading control. The protein levels of misfolded α-syn (**D**) and p-α-syn (**F**) were normalized to those of β-actin. (*p < 0.05, **p < 0.01, ****p < 0.0001, *n* = 3/group). **G**–**J** Primary neurons at DIV6 were transduced with LVs containing *Flag*-*KAT8* or *Flag*. PBS or 1 µg/mL PFFs were added to the neurons at 7 DIV for 14 days. Representative images of immunofluorescence staining for p-α-syn and Map2 in (**G**). Scale bar, 10 μm. Quantification of p-α-syn intensity in (**H**) (*p < 0.05, *n* = 5/group). The lysosomal-related protein levels were visualized by immunoblotting in (**I**), and the quantified protein levels were normalized to those of β-actin in (**J**). (*p < 0.05, **p < 0.01, *n* = 3/group). The data are presented as the means ± SEMs. Statistical significance was determined by an unpaired *t*-test in (**J**) or two-way ANOVA in (**B**), (**E**), (**F**), and (**H**).

To identify the effects of KAT8 on α-syn pathology, we inactivated KAT8 via MC4033 in primary neurons that were treated with α-syn PFFs. *GBA1* overexpression repressed α-syn aggregation induced by α-syn PFFs; however, inactivation of KAT8 by MC4033 abolished the *GBA1* overexpression-induced repressive effects on α-syn aggregation (Fig. 4A, B). We next examined whether KAT8 overexpression influences α-syn aggregation. We used LV vectors encoding KAT8 to increase its expression in primary neurons. KAT8 overexpression ameliorated α-syn PFF-induced α-syn aggregation (Fig. 4C, D) and pathological α-syn formation (Fig. 4E, F). Moreover, KAT8 overexpression alleviated pathological α-syn aggregation, as indicated by pS129 α-syn staining (Fig. 4G, H). We further examined the effects of KAT8 overexpression on cathepsin levels. In primary neurons in which KAT8 was overexpressed, the expression of cathepsins CTSB, CTSD, and CTSL increased (Fig. 4I, J), indicating that KAT8-driven lysosomal biogenesis contributed to the reduction in pathological α-syn aggregation.

### GBA1 attenuates α-syn pathology in an α-syn PFF mouse model

**Fig. 5:**
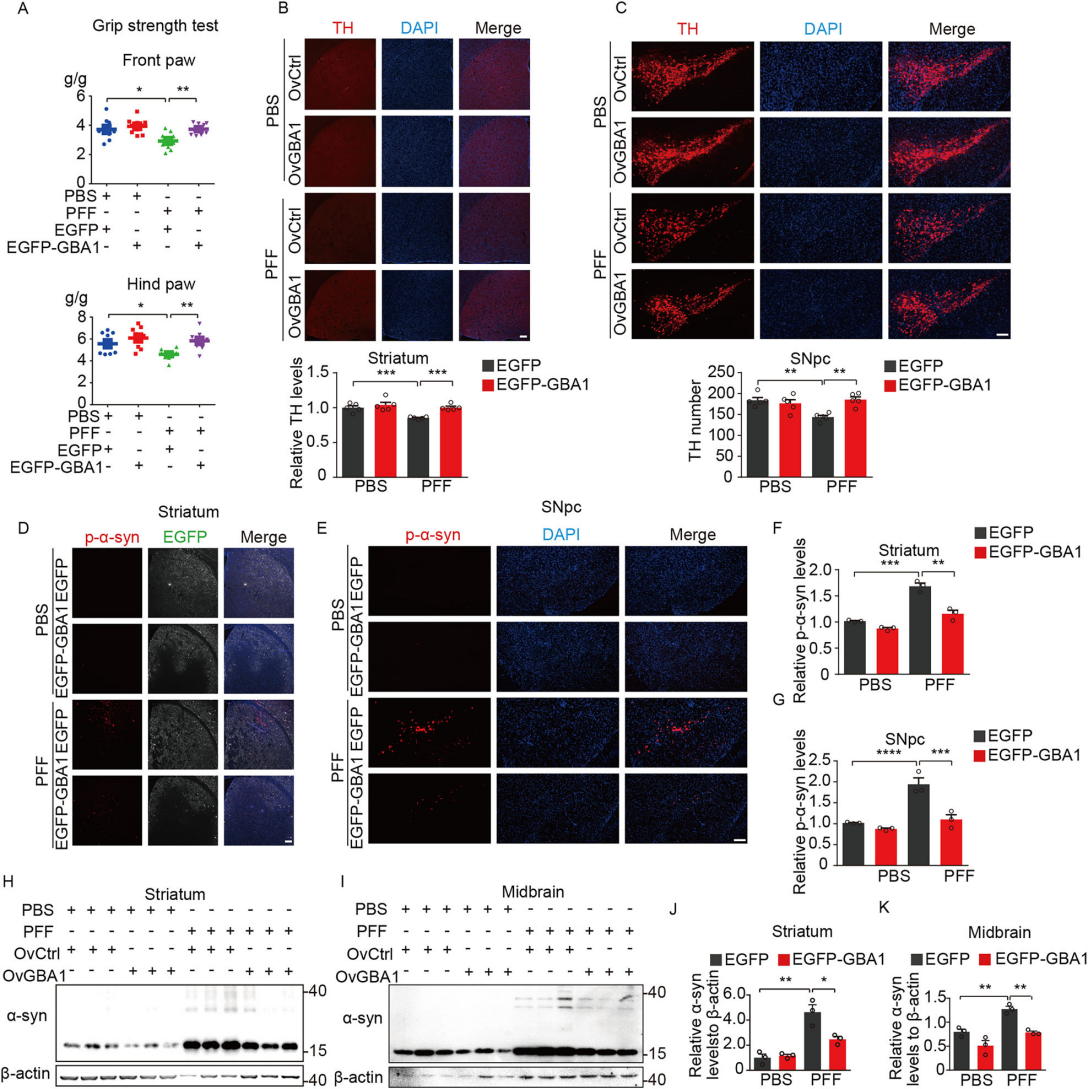
Overexpression of GBA1 rescues α-syn aggregation and degeneration pathology in an α-syn PFF mouse model. The mice received injections of AAV-*EGFP*-*GBA1* (2E10 vg/mouse) or AAV-*EGFP* (2E10 vg/mouse) in the bilateral dorsal striatum, followed by 2.5 µg/side PFFs or PBS 1 month later. Behavioral testing and histological analysis were performed 3 months after the injection of PFFs. **A** Quantification of grip strength normalized to mouse weight (g/g). (*p < 0.05, **p < 0.01, *n* = 10/group). **B** Representative images of staining for TH in the striatum. Scale bar, 100 μm (the top). The percentage of the area covered by TH staining was quantified. (the bottom). (***p < 0.001, *n* = 3/group). **C** Representative images of TH staining in the SNpc. Scale bar, 100 μm (the top). Quantification of TH-positive cells in the SNpc of mice (the bottom) (**p < 0.01, *n* = 3/group). Representative images of p-α-syn staining in the striatum (**D**) and SNpc (**E**) with quantification of the percentage of the area covered by p-α-syn staining in the striatum (**F**) and SNpc (**G**). Scale bar, 100 μm. (**p < 0.01, ***p < 0.001, ****p < 0.0001, *n* = 5/group). Western blot analysis of TX-insoluble and SDS-soluble misfolded α-syn protein levels in the striatum (**H**) and midbrain (**I**) with quantification of misfolded α-syn protein levels in the striatum (**J**) and SNpc (**K**). Statistical significance was determined by two-way ANOVA in (**A**–**C**), (**F**), (**G**), (**J**) and (**K**).

Given that *GBA1* overexpression enhances lysosomal biogenesis and reduces α-syn aggregation in vitro, we next determined whether *GBA1* overexpression could mitigate α-syn pathology in vivo in an α-syn PFF mouse model. Using an AAV-mediated gene delivery strategy with AAV-EGFP-*GBA1* (or AAV-EGFP as a control), widespread expression of *GBA1* was observed four months after the AAVs were injected into the striatum in adult mice (Fig. S3A–D). After a 3-month course of a-syn PFF injection, the forelimb grip strength significantly decreased in the mice (Fig. 5A, B). However, in the α-syn PFF mice in which *GBA1* was overexpressed, the grip strength significantly improved (Fig. 5A). Moreover, TH immunoreactivity staining revealed that *GBA1* overexpression rescued the loss of dopaminergic neurons in α-syn PFF model mice, as evidenced by the increased fluorescence intensity of TH-positive areas in the striatum and increased number of TH-positive neurons in the SNpc (Fig. 5B, C). Furthermore, *GBA1* overexpression significantly decreased the number of pathological pS129 α-syn aggregates in the striatum and SNpc, as assessed by immunofluorescence staining for pS129 α-syn (Fig. 5D–G). In addition, *GBA1* overexpression significantly decreased the levels of TX-insoluble and SDS-soluble α-syn species in the striatum and midbrain of α-syn PFF mice (Fig. 5H to K).

**Fig. 6:**
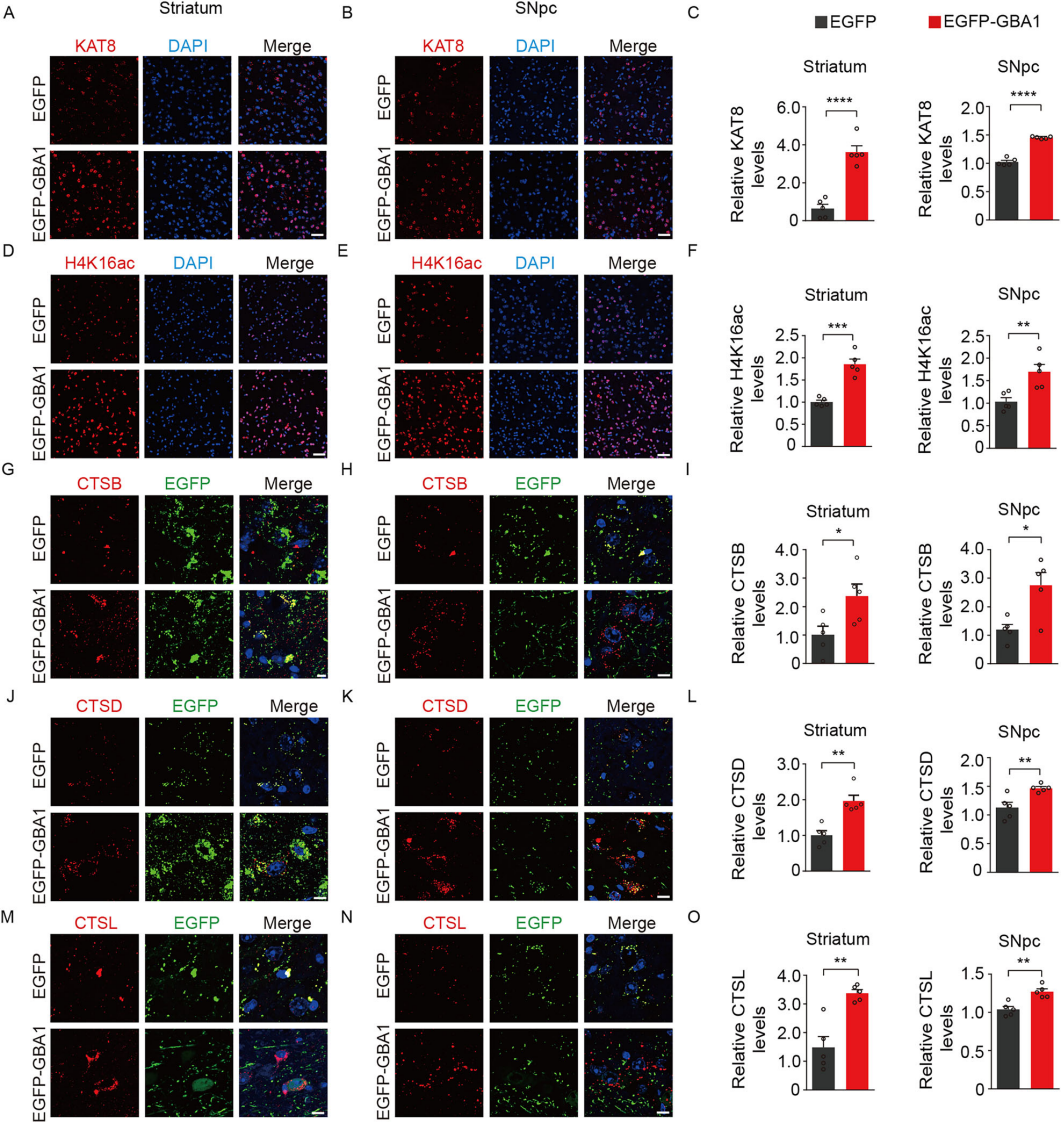
Overexpression of GBA1 increased KAT8 and H4K16ac protein levels, as well as lysosomal hydrolase expression, in a mouse model. The mice received injections of AAV-*EGFP*-*GBA1* (2E10 vg/mouse) or AAV-*EGFP* (2E10 vg/mouse) in the bilateral dorsal striatum for 4 months. Representative images of KAT8 (**A, B**) and H4K16ac (**D, E**) staining in the Striatum (left) and SNpc (right). Scale bar, 25 μm. The quantification of the percentage of the area covered by KAT8 (**C**) and H4K16ac (**F**) staining in the striatum (left) and SNpc (right). (*p < 0.05, ***p < 0.001, ****p < 0.0001, *n* = 5/group). Representative images of cathepsin CTSB (**G**, **H**), CTSD (**J**, **K**) and CTSL (**M**, **N**) staining in the striatum (left) and SNpc (right). Scale bar, 25 μm. The quantification of the percentage of the area covered by cathepsin CTSB (**I**), CTSD (**L**), and CTSL (**O**) staining in the striatum (left) and SNpc (right). (*p < 0.05, **p < 0.01, ***p < 0.001, ****p < 0.0001, *n* = 5/group). The data are presented as the means ± SEMs. Statistical significance was determined by an unpaired *t* test in (**C**), (**F**), (**I**), (**L**) and (**O**).

We then examined the effects of *GBA1* overexpression on KAT8 expression and lysosomal biogenesis. *GBA1* overexpression resulted in increased levels of KAT8 in the striatum and midbrain (Figs. 6A–C, S3C, D). Moreover, immunofluorescence staining revealed increased H4K16ac expression, suggesting that *GBA1* overexpression induces KAT8-mediated histone acetylation in vivo (Fig. 6D–F). Moreover, in the mice in which *GBA1* was overexpressed, increases in the expression of the lysosomal cathepsins CTSB, CTSD, and CTSL were observed in the striatum and midbrain, as indicated by immunofluorescence staining (Fig. 6G–O) and Western blotting (Fig. S3E–H). Collectively, these findings further suggest that *GBA1* overexpression reduces α-syn aggregation pathology, which is mediated by KAT8-driven lysosomal biogenesis, in an α-syn PFF mouse model.

## Discussion

Variants in the *GBA1* gene are associated with reduced lysosomal glucocerebrosidase activity and elevated risk of Parkinson’s disease. Notably, in specific populations, 70-80% of PD-associated *GBA1* variants are the missense mutations c.1448 T > C (L444P) and c.1226 A > G (N370S) [18]. GCase deficiency leads to autophagic dysfunction, lysosomal enlargement, and impaired autophagy-lysosome reformation (ALR) [42, 43]. Misfolded GCase further blocks the multimerization of LAMP2A, thereby inhibiting chaperone-mediated autophagy (CMA) and impairing the degradation of key substrates such as monomer α-syn [44]. Moreover, in human iPSC‑derived dopaminergic neurons, GBA1 mutations exert variant‑specific disruptive effects on proteostasis, thereby contributing to the α-syn aggregation [45]. Furthermore, GBA1 dysfunction leads to the accumulation of unhydrolyzed lipid substrates, which compromise lysosomal degradative capacity and promote α-syn aggregation, ultimately enhancing PD susceptibility [17, 46]. Our findings demonstrate that KAT8 mediates GBA1-regulated lysosomal enzyme activity that promotes α-syn degradation and the autophagy-lysosomal pathway, suggesting that KAT8 may be functionally involved in those pathways. Collectively, these findings highlight the central role of GCase deficiency in PD pathogenesis, driven by *GBA1* variants. Consequently, innovative therapeutic strategies targeting the restoration of neuronal GCase activity represent a promising and translationally relevant approach for PD intervention. In this study, we elucidated the mechanistic connections among GCase activity, lysosomal homeostasis, and α-syn pathology. Using complementary approaches involving lentiviral-mediated GBA1 overexpression in primary neurons and AAV-mediated gene delivery in mice, we demonstrated that GCase activation mitigated α-syn PFF-induced neurodegeneration through enhancing lysosomal function. Mechanistically, we identified KAT8-mediated histone acetylation as a novel regulatory axis through which GCase modulates lysosomal biogenesis. Importantly, genetic activation of KAT8 recapitulates the protective effects against α-syn aggregation observed with GBA1 overexpression. In vivo validation in α-syn PFF-injected mice revealed that GBA1 augmentation not only reduces α-syn pathology and dopaminergic neurodegeneration but also improves motor performance, paralleled by increased KAT8-associated histone acetylation and lysosomal biogenesis. These findings establish an epigenetic mechanism underlying GCase-mediated neuroprotection and provide mechanistic insights for developing targeted PD therapies. Although our data support a GCase activity-dependent link among GBA1 function, KAT8-mediated H4K16 acetylation, and lysosomal gene expression, the effects of this signaling axis in GBA1 heterozygous mutant cell or animal models remain to be validated. Identification of these links using disease-relevant genetic models, including knock-in animals or patient-derived iPSCs, will help determine the extent to which KAT8-dependent chromatin regulation is engaged in the context of PD-associated GBA1 mutations.

Pathological α-syn aggregation in neurons induces lysosomal dysfunction [47]. The structural characteristics of α-syn aggregates confer a strong affinity for membrane binding, leading to tight association with the lysosomal membrane. This association likely contributes to membrane disruption and damage. Lysosomes typically maintain an acidic luminal pH (pH 4.5–5.0) through the action of the proton-pumping V-type ATPase (V ATPase) on the lysosomal membrane. This acidic environment is crucial for the activation of hydrolytic enzymes during proteolysis. Disruption of lysosomal membranes can alter lysosomal pH through increased membrane permeabilization or by impairing the proper positioning of lysosomal V-ATPase, resulting in decreased activity of lysosomal hydrolases and impaired degradation of α-syn. Consistent with recent literature, our findings demonstrate that α-syn aggregates reduce GCase activity in neurons [48]. Notably, both GCase activity and protein levels are diminished in specific brain regions of idiopathic PD patients without GBA1 mutations [15, 49]. The overexpression of *GBA1* or the use of GCase activators prevents α-synucleinopathy in PD animal models [50, 51]. However, the specific molecular mechanisms by which GBA1 regulates α-syn-mediated pathology remain to be elucidated.

**Fig. 7:**
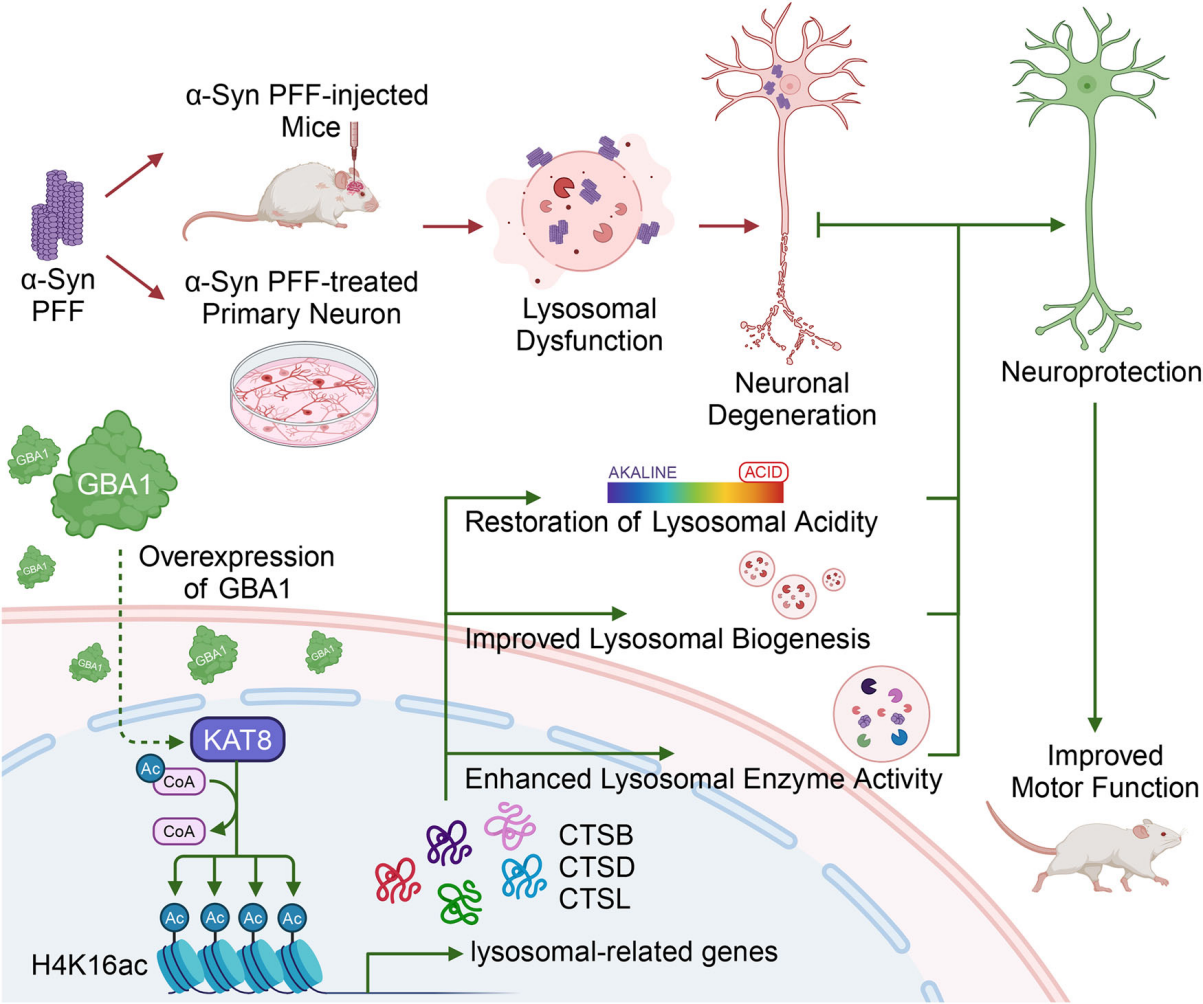
KAT8 functions in a GBA1-mediated therapeutic model for α-syn pathology. α-Syn PFFs induce pathological accumulation of endogenous α-syn and lysosomal dysfunction, which causes irreversible damage to neurons. *GBA1*, encoding a crucial lysosomal hydrolase, represents the most significant genetic risk factor for PD. This enzyme plays a pivotal role in the lysosome-mediated degradation of pathological proteins. In addition to the established α-synuclein-targeted therapeutic strategies involving GBA1-overexpressing viral vectors, our study reveals a novel mechanism underlying the neuroprotective effects of GBA1 in PD treatment. Specifically, we demonstrated that GBA1 upregulates the expression of KAT8, a histone acetyltransferase. This upregulation leads to increased acetylation of histone H4 at lysine 16, subsequently initiating the transcriptional activation of genes encoding lysosomal degradation-related proteins, including cathepsin B, cathepsin D, and cathepsin L. These cathepsin family members are critically involved in the lysosomal clearance of pathological α-synuclein aggregates, thereby providing a molecular basis for GBA1-mediated neuroprotection in PD. Created with BioRender.com.

Previous studies have demonstrated that increased glucocerebrosidase activity can improve lysosomal function and positively influence α-synuclein clearance [50, 52]. These observations are in agreement with our findings; beyond that, our study offers novel insights into the molecular mechanisms by which GBA1 enhances lysosomal activity. Specifically, GBA1 upregulates KAT8 expression, which induces KAT8-driven histone acetylation, resulting in the activation of lysosomal gene expression (Fig. 7). TFEB is recognized as the master regulator of lysosomal biogenesis [53], which translocates into the nucleus to induce the expression of multiple lysosome-associated genes [32, 34, 50, 54]. Our data reveal a TFEB-independent pathway in which GBA1 upregulates H4K16 acetylation to activate the transcription of lysosomal genes. Gene expression is regulated not only by transcription but also by chromatin modifications [55, 56]. Several epigenetic regulatory mechanisms, such as DNA methylation, histone posttranslational modifications, and chromatin remodeling, have been demonstrated to modulate chromatin structure and regulate a broad spectrum of cellular and biological processes. Histone acetylation directly promotes the formation of an open chromatin configuration [57], and its dynamic regulation critically influences lysosomal biogenesis and autophagy processes [35, 58–60]. In Alzheimer’s disease (AD) and PD, the reduction in histone H3 and H4 acetylation induces transcriptional silencing, thereby leading to diminished expression of neurotrophins. Additionally, decreased acetylation of H3 and H4 is associated with increased accumulation of β-amyloid (Aβ) plaques, neurofibrillary tangles (NFTs), and Lewy bodies (LBs) [61]. These results suggest that the regulation of histone acetylation is important for the treatment of PD.

Histone acetylation can promote the expression of lysosomal and autophagy-related genes [35, 62], and the regulation of histone acetylation by histone acetyltransferases (HATs) and histone deacetylases (HDACs) fundamentally modulates the development of neurodegenerative diseases [63, 64]. To investigate the role of GBA1 in regulating the expression of lysosomal-related genes, we examined HATs family mRNA levels after GBA1 overexpression in neurons and found that *KAT8* was significantly regulated by GBA1, and further analysis of protein levels revealed that GBA1 promoted KAT8 expression. *KAT8* has been identified as a candidate gene for PD by genome-wide studies and PINK1-mitophagy screening [12, 65]. KAT8 plays a crucial role in the transcriptional activation of genes associated with PD, such as *CTSB* and *PINK1*. *KAT8* encodes a component of a nonspecific lethal histone acetylation complex that is localized to the nucleus, where it acetylates histone H4 on lysine 16 (H4K16ac) to regulate autophagy [54]. Additionally, it is involved in the regulation of lysosomal and mitochondrial gene transcription [65–70]. Our results further demonstrated that the overexpression of KAT8 in neurons not only activated the transcription of lysosome-related genes but also significantly alleviated the pathological accumulation of α-syn. Overall, these findings reveal a potentially wider role for KAT8 in regulating genes and pathways implicated in PD.

In our study, inhibition of KAT8 by the selective inhibitor MC4033 suppressed the GBA1-mediated epigenetic modifications involved in lysosomal biogenesis. Notably, pharmacological inhibition of KAT8 abolished these protective effects, confirming its essential role in GBA1-mediated lysosomal activation. These findings further indicate that GBA1 not only functions as an enzyme influencing sphingolipid metabolism and enhancing lysosomal function but also plays a role in regulating histone modification. We also investigated whether other autophagy-related KATs (KAT5 and KAT7) are involved in GBA1-mediated lysosomal biogenesis [33, 62]. Inhibition of KAT5 or KAT7 produced weaker and more limited effects on lysosomal proteins compared with KAT8, potentially attributed to overlapping histone modification sites among these KATs [71]. Moreover, our data indicate that KAT8 is a major KAT in mediating the regulation of lysosomal capacity by GBA1, as KAT5 and KAT7 only have limited or gene-restricted effects. Nevertheless, the precise mechanism by which GBA1 influences KAT8-driven histone acetylation and modulates PD pathology requires further investigation.

These results suggest that the regulation of histone acetylation is important for the treatment of PD. Importantly, our research results indicate that GBA1 enhances α-syn clearance through epigenetic reprogramming. Both in vitro and in vivo models consistently demonstrate that GBA1 overexpression (1) alleviates motor deficits, (2) reduces α-syn pathology in the nigrostriatal circuit, and (3) is associated with increased KAT8/H4K16ac signaling and cathepsin activation. Our findings identify GBA1 as a major regulator of this epigenetic landscape. These converging lines of evidence strongly suggest that targeting GBA1-mediated epigenetics and the histone acetylation pathway represents a paradigm-shifting approach with disease-modifying potential in PD.

These findings provide valuable insight into the molecular mechanisms by which GBA1 overexpression enhances lysosomal function and inhibits α-syn aggregation through mitigating neurodegeneration caused by pathological α-syn aggregates. Understanding these mechanisms paves the way for the development of neuroprotective therapies targeting α-synucleinopathies in PD. Additionally, strategies aimed at regulating specific histone acetylations to promote lysosomal biogenesis may offer promising therapeutic avenues for PD and other neurodegenerative diseases.

## Materials and Methods

### Animals

Male C57BL/6J mice were purchased from Shanghai SLAC Laboratory Animals (Shanghai, China). All the mice were housed under a 12 h light‒dark cycle at 23 °C in a pathogen-free barrier facility. The animal experiments were approved by the Animal Committee of Soochow University. For treatment, all the animals were randomly divided into four groups: (1) the PBS + AAV-EGFP group; (2) the PFF + AAV-EGFP group; (3) the PBS + AAV-EGFP-*GBA1* group; and (4) the PFF + AAV-EGFP-*GBA1* group. The mice were administered AAV and injected with α-syn PFFs one month later. The mice were injected bilaterally into the striatum (coordinates: AP + 0.2 mm, ML ± 2.0 mm, DV -2.6 mm) with 2.5 μg/side α-synuclein PFFs or PBS. The final titers of the injected solutions were 2E10 vg/mouse (2 μL/side) for AAV-EGFP and AAV-EGFP-*GBA1* (Genechem, China). Three months after PFF injection, the mice were perfused transcardially with PBS, followed by 4% paraformaldehyde (PFA). The brains were removed and incubated overnight in 4% PFA.

### Reagents and antibodies

ACSS2-IN (Ac-CoA synthase inhibitor 1) (HY-104032), MC4033 (HY-149302), and leupeptin hemisulfate (HY-18234A) were purchased from MedChemExpress (New Jersey, USA). NU 9056 (TargetMol, T23095) and WM-3835 (TargetMol, T9102) were obtained from TargetMol (Boston, Massachusetts, USA). Antibodies against 4′,6-diamidino2-phenylindole (DAPI) (#28718-90-3) and anti-β-actin (A1978) were purchased from MedChemExpress Sigma-Aldrich (St. Louis, MO, USA). Antibodies against anti-α-synuclein (phospho S129) (ab51253), anti-Galectin-3 (ab76245), GCase (ab128879), anti-NeuN (ab177487), and anti-KAT8 (ab109463) were purchased from Abcam (Cambridge, UK). Antibodies against anti-Cathepsin B (#12216-1-AP), anti-Cathepsin D (#21327-1-AP), anti-Cathepsin L (#27952-1-AP), and TH (#25859-1-AP) were purchased from ProteinTech (Wuhan, China). Antibodies against anti-α-synuclein (#610787) were purchased from BD Biosciences (San Jose, California, USA). Antibodies against anti-Histone H3 (AF0009) and anti-Acetyl-Histone H4 (Lys16) (AG4204) were purchased from Beyotime Biotechnology (Shanghai, China). Antibodies against donkey anti-mouse IgG (H + L) Alexa Fluor 488 (A21202), donkey anti-mouse IgG (H + L) Alexa Fluor 568 (A11004), donkey anti-rabbit IgG (H + L) Alexa Fluor 488 (A21206), and donkey anti-rabbit IgG (H + L) Alexa Fluor 568 (A11011) were purchased from Thermo Fisher Scientific (Waltham, Massachusetts, USA). Antibodies against goat anti-mouse immunoglobulin G (IgG) (H + L)–HRP (#115-035-008) and goat anti-rabbit IgG (H + L)-HRP (111-035-003) were purchased from Jackson ImmunoResearch (West Grove, Pennsylvania, USA). Anti-MAP2 was purchased from Chemicon (Temecula, California, USA).

### Cell culture

Primary cortical neuron cultures were generated from E16.5 mice as described previously [29]. Dissociated cortical neurons were seeded on plates that were coated with poly-D-lysine (Sigma‒Aldrich, P7886) and cultured in Neurobasal medium (Invitrogen, 21103049) supplemented with B-27 (Invitrogen, 17504044), GlutaMAX (Invitrogen, 35050061), penicillin (100 μg/ml), and streptomycin (100 μg/ml) (Invitrogen, 15070063). HEK293 cells and HEK293T/17 cells were obtained from ATCC (Manassas, VA, USA) and grown in Dulbecco’s modified Eagle’s medium (DMEM) supplemented with 10% heat-inactivated fetal bovine serum (FBS), penicillin (100 μg/mL), and streptomycin (100 μg/mL) (Invitrogen, 15070063) in a humidified incubator at 37 °C with 5% CO2. Human bone osteosarcoma epithelial cells (U2OS) were obtained from ATCC (Manassas, VA, USA) and grown in DMEM supplemented with 10% FBS, penicillin (100 μg/ml), streptomycin (100 μg/ml), and 1 M HEPES.

### Lysosomal function assays

Primary cells were treated with 1 μg/ml PFFs for 14 days to induce lysosomal damage. The cells were fixed with 4% PFA for 5 minutes, incubated with anti-Galectin-3 overnight, incubated with Alexa Fluor secondary antibodies (Thermo Fisher Science, Waltham, MA, USA) for 2 h, and observed via confocal microscopy. To determine changes in lysosomal pH, primary cells were incubated with 1 μM LysoTracker for 30 min in a CO2 incubator at 37 °C, washed with PBS, incubated with Hoechst, and observed via confocal microscopy.

The DQ-BSA dye was purchased from Share-bio (D-12051SB, Shanghai, China) for use in the hydrolase activity assays. DQ-BSA was incubated with the cells at a final concentration of 50 µg/ml in prewarmed medium for 8 h. After treatment, the cells were washed three times in warmed PBS and then fixed in 4% PFA for 5 min. After fixation, the cells were washed three times with PBS and then stained with DAPI for 10 min. DQ-BSA fluorescence was visualized via confocal microscopy. Quantification was conducted with ImageJ software (version 1.41; National Institutes of Health) as follows: a density threshold was established to measure positive staining based on the corresponding negative control. The threshold was selected to exclude nonspecific background staining. The same thresholds and system settings were applied to all the slides. The number of pixels within the threshold, indicating the quantity of positive staining, was recorded for each area.

### ChIP‒qPCR assay

ChIP assays were performed via the Enzymatic Chromatin IP Kit (SimpleChIP® Enzymatic Chromatin IP Kit, CST, 9003). Sonicated chromatin extracts were incubated with antibodies against H4K16ac. The bound DNA sequences were determined via quantitative real-time PCR with the primer sequences listed in the supplementary table.

The ChIP‒qPCR values were normalized to those of the input control and are presented as the fold enrichment relative to the anti-normal IgG control.

### Plasmid transfection

The Flag-GBA1 plasmid (no. 74658) was purchased from Addgene (Watertown, Massachusetts, USA). The Flag-*GBA1*^P370S^ and Flag-*GBA1*^L444P^ plasmids were synthesized and verified by sequencing by Langjing Biology (Shanghai, China). U2OS cells were transfected with plasmids using Lipofectamine 2000 (Invitrogen, Carlsbad, California, USA) for 48 h. The cells were then collected for further ChIP assays. For stable expression of *GBA1* in this study, the DNA sequence of *GBA1* was inserted into pLVX-AcGFP1-C1 (no. 632155, Clontech, Mountain View, California, USA) to express EGFP-*GBA1*. Additionally, *GBA1* was inserted into pCDH-EF1-FHC (no. 64874, Addgene, Watertown, Massachusetts, USA) to express Flag-*GBA1*. The Flag-KAT8 plasmid (SP-1759) was purchased from the Research Innovation Center (Suzhou, Jiangsu Province, China). *KAT8* was also inserted into pCDH-EF1-FHC to express Flag-KAT8 in primary neurons.

### Immunofluorescence staining

The primary neurons were first fixed with 4% PFA for 5 min and then permeabilized with 0.25% Triton X-100 for another 5 min. After three washes with PBS, the cells were blocked for 10 min with 2% FBS in PBS. Next, the cells were incubated with antibodies at 4 °C overnight, followed by incubation with Alexa Fluor secondary antibodies (Thermo Fisher Scientific, Waltham, MA, USA) for 2 h at room temperature. The cells were subsequently stained with DAPI for 5 min and then washed three times with PBS. Finally, the cells were observed with a confocal microscope system.

### Real-time PCR (qPCR)

Total mRNA was extracted via TRIzol reagent following the manufacturer’s protocol (Invitrogen, 15596026). The RNA concentration was measured via a NanoDrop 2000 (Thermo Fisher Scientific). Subsequently, 500 ng of total mRNA was used to produce cDNA via HiScript III RT SuperMix (R323-01, Vazyme Biotech, Nanjing, Jiangsu Province, China). For the qPCR, ChamQ Universal SYBR qPCR Master Mix (Q711-02, Vazyme Biotech, Nanjing, Jiangsu Province, China) and an ABI-7500 Fast Real-Time PCR system (Applied Biosystems, Waltham, Massachusetts, USA) were utilized. The sequences of the primers are summarized in the supplementary table.

### Immunoblotting

Mouse brain tissues and cultured cell samples were homogenized and lysed in lysis buffer (1 M Tris-HCl, pH 7.6, 1% NP-40, 5% sodium deoxycholate, 1 M NaCl, and 1× protease inhibitor mixture) and diluted in 2× sample buffer (1 M Tris-HCl, pH 6.8, 10% SDS, 4% (v/v) glycerol, 2% (v/v) 2-mercaptoethanol, and 0.01% (w/v) bromophenol blue). Equal amounts of protein were separated via SDS‒PAGE and transferred to polyvinylidene difluoride (PVDF) membranes (Millipore, Burlington, Massachusetts, USA). The membranes were blocked in 5% skim milk in TBST for 1 h at room temperature. Next, the membranes were incubated with primary antibodies in Tris-buffered saline supplemented with 0.1% Tween 20 (TBST) at 4 °C overnight. Afterward, the membranes were incubated with an HRP-conjugated IgG secondary antibody for 2 h at room temperature. The proteins on the membranes were detected via an enhanced chemiluminescence (ECL) detection kit (P10300, NCM Biotech, Nanjing, Jiangsu Province, China).

### Immunostaining

For immunostaining of mouse brain sections, the brains were collected after perfusion with 4% PFA, fixed in 4% PFA for 3 days at 4 °C, and then dehydrated with 30% sucrose for another 3 days at 4 °C. Twenty-micrometer-thick serial brain sections were collected and blocked with 5% FBS in PBS supplemented with 1% Triton X-100 for 1 h at room temperature, followed by overnight incubation with primary antibodies at 4 °C. After primary antibody incubation, the sections were washed three times in PBS and incubated with a mixture of Alexa Fluor 488- and Alexa Fluor 568–conjugated secondary antibodies for 1 h at room temperature, followed by DAPI staining for 10 min at room temperature. The fluorescence images were captured via confocal microscopy (LSM710, Zeiss, Jena, Germany, and AIR HD25, Nikon, Tokyo, Japan).

For immunostaining of cultured primary cells, the cells were cultured on glass coverslips and fixed with 4% PFA for 5 min. After fixation, permeabilization was performed with 0.1% Triton X-100 in PBS, and the cells were blocked with 1% FBS in PBS, followed by incubation with primary antibodies at 4 °C overnight. The next day, the coverslips were washed three times in PBS, incubated with a fluorescence-conjugated secondary antibody for 1 h at room temperature, and then stained with DAPI for 10 min at room temperature. The slides were imaged via confocal microscopy (LSM710, Zeiss, Jena, Germany, and AIR HD25, Nikon, Tokyo, Japan).

### Cell cytotoxicity assay

The cytotoxicity of primary neurons was detected via the CytoTox 96 Non-Radioactive Cytotoxicity Assay (Promega, Madison, WI, USA). In brief, 50 µL of growth medium collected from primary neuron cells was mixed with 50 µL of CellTiter-Go at room temperature for 20 min. The absorbance was detected at 490 nm by a microplate reader (Infinite M1000 Pro, Tecan Group Ltd., Männedorf, Switzerland) to determine cell cytotoxicity.

### GCase activity assay

The cells were washed twice with PBS and scraped in PBS supplemented with protease and phosphatase inhibitors. The cells were spun down at 10,000×*g* for 10 min, and the supernatant was removed. The cell pellets were suspended in ice-cold activity assay buffer (citrate-phosphate buffer, pH 5.4; 0.25% Triton-100; and 1 mM EDTA) supplemented with protease and phosphatase inhibitors. The cells were then sonicated 20 times for 1 second at a setting of 2.5 (QSonica Microson XL-2000) and rotated for 15 minutes at 4 °C to allow for full resuspension of the proteins. The samples were centrifuged for 15 min at 10,000×*g*, and the supernatant was collected. The lysates were then incubated for 30 minutes. The samples were incubated with 10 mM 4-methylumbelliferyl β-D-glucopyranoside (Sigma‒Aldrich, M3633) in activity assay buffer for 1 h at 37 °C. One volume of stop buffer (1 M glycine, pH 12.5) was added, and the plates were read on a spectrophotometer (lex = 365, lem = 445). All fluorescence readings are expressed in relative fluorescence units (RFU), and GCase activity was described as the RFU per mg of protein sample.

### Cathepsin B/L and cathepsin D/E activity assays

Cathepsin B/L and D/E activities were measured via the following fluorogenic substrates: 200 µM Z-Arg-Arg-AMC (Sigma‒Aldrich, C5429) for cathepsin B/L and 20 µM Z-RR-AMC (MedChemExpress, HY-P2498A) for cathepsin D/E. The reaction was carried out in 100 µL of activation buffer (Tris-HCl, pH 5.0, 0.2% Triton X-100, 1 mM EDTA, 5 mM DTT) and incubated at 37 °C for 1 h. Fluorescence was recorded as relative fluorescence units (RFU): cathepsin B/L at 328 nm (excitation) and 393 nm (emission), and cathepsin D/E at 355 nm (excitation) and 460 nm (emission). The activities are expressed as RFU per mg of protein.

### Behavioral tests

All tests were conducted between 9:00 and 15:00 during the light cycle. The mice were subjected to the pole test, and the equipment was cleaned with 30% ethanol after each animal. The grip test was used to evaluate the maximum muscle strength of all four limbs. A grip meter (Bio-GT3, BIOSEB, Vitrolles, France) attached to a force transducer was used to measure the peak force produced. The mice were positioned on a 10×10 cm grid with their front paws (2 paws) or back paws (2 paws) on the grid and were gently pulled backward until their grip was released. Three trials were conducted, and the results were averaged. The results were normalized to the body weight (g/g, relative grip strength).

### Randomization and blinding procedures

Randomization was performed using a random number table. Briefly, all animal experiments were conducted and analyzed in a blinded fashion. Mice within a predefined weight range were initially assigned temporary random numbers, and following random group allocation, permanent numerical identifiers were assigned to each mouse and labeled on their cages. To minimize experimental bias, cages were randomly selected from the total cohort during data collection. Both data acquisition and subsequent statistical analysis were performed by two independent investigators who were masked to the group assignments and treatment regimens.

### Statistical analysis

The quantification of the immunoblots was performed via Photoshop 7.0 (Adobe, San Jose, CA, USA). GraphPad Prism 7.00 (GraphPad Software, version X; La Jolla, CA, USA) was used for statistical analysis and graphing. Significant differences were evaluated via unpaired t tests or two-way analysis of variance (ANOVA) followed by Dunnett’s or Tukey’s multiple comparisons tests. The criterion of significance was set at *p < 0.05, **p < 0.01, and ***p < 0.001; “ns” indicates no significant difference. The values are shown as the means ± SEMs (standard error of the mean).

## Data and materials availability

All the data are available in the main text or the supplementary materials.

## Supplementary information

Supplementary text

Figure S1

Figure S2

Figure S3

Original Data

Supplementary table
