# Supplementary material for: Lysine acetyltransferase 8-mediated histone acetylation, regulated by GBA1, is associated with lysosomal function related to α-Synuclein pathology
Source: Cell Death Dis. 2026 Jun 8;17(1):711. doi: 10.1038/s41419-026-08928-2 (PMC13462883; doi:10.1038/s41419-026-08928-2)
Supplement: Supplementary file 1 [file 41419_2026_8928_MOESM5_ESM.pdf]

Figure 2 F

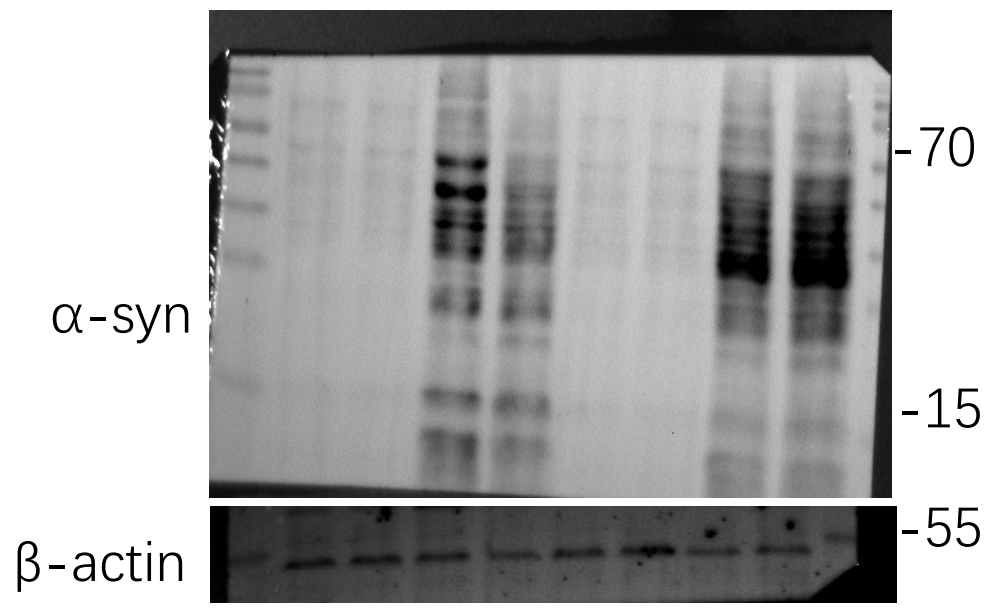

Figure 2 I

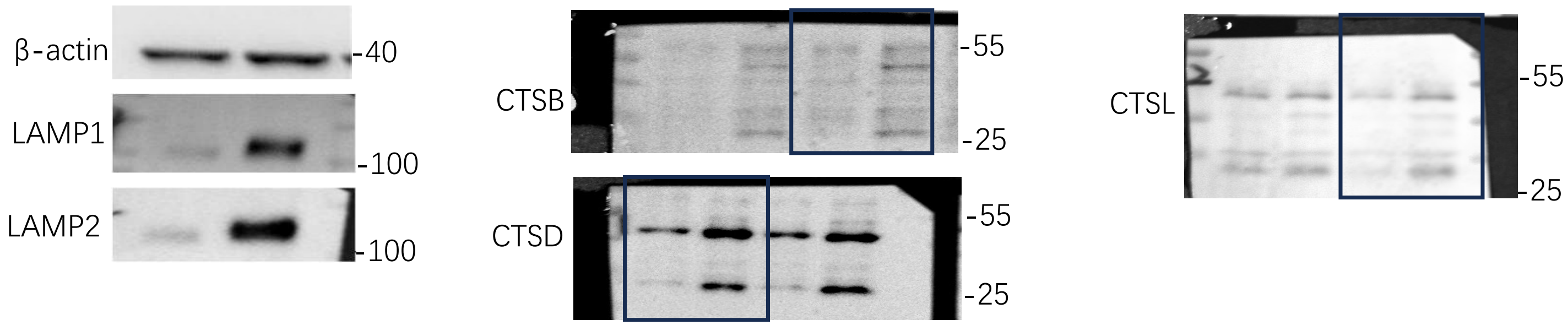

Figure 3 B

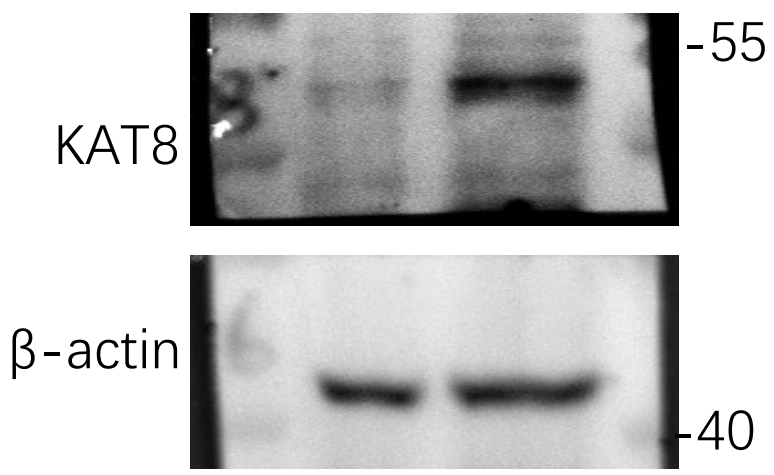

Figure 3 D

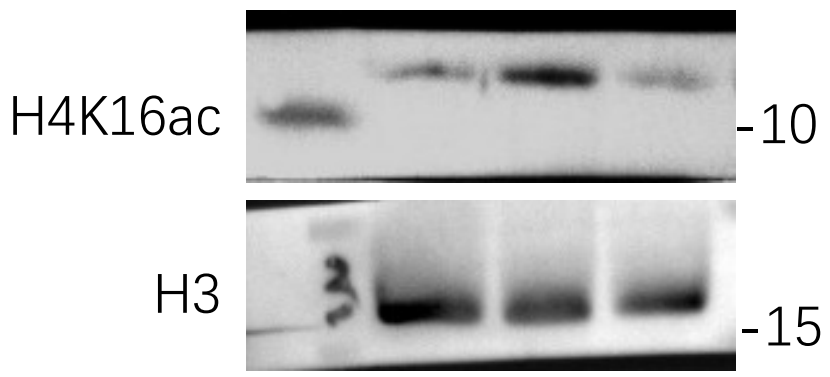

Figure 3 F

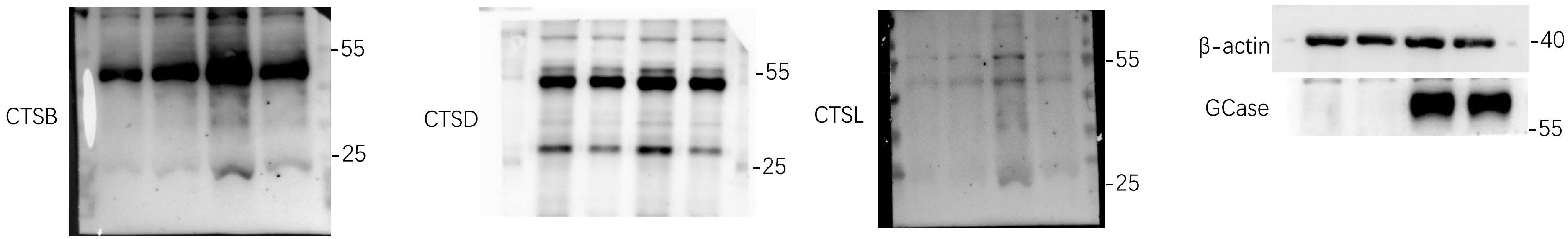

Figure 3 I

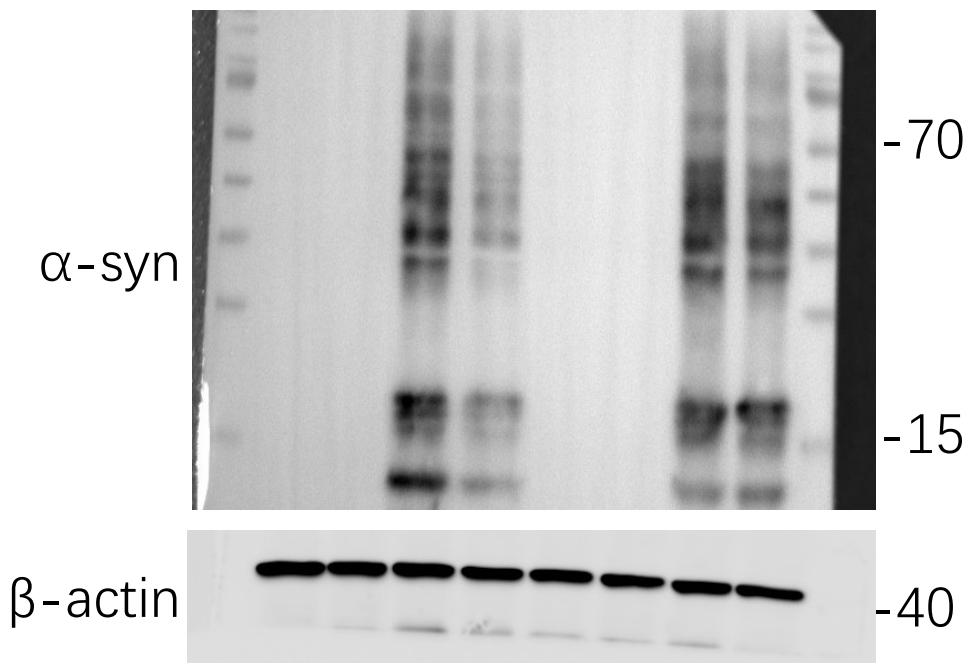

Figure 4 A

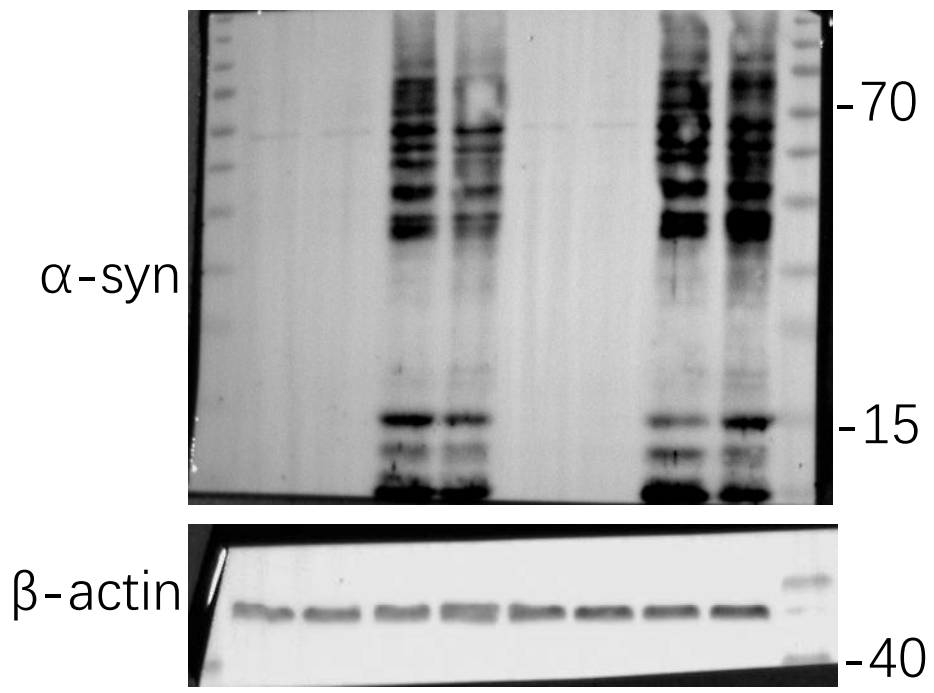

Figure 4 C

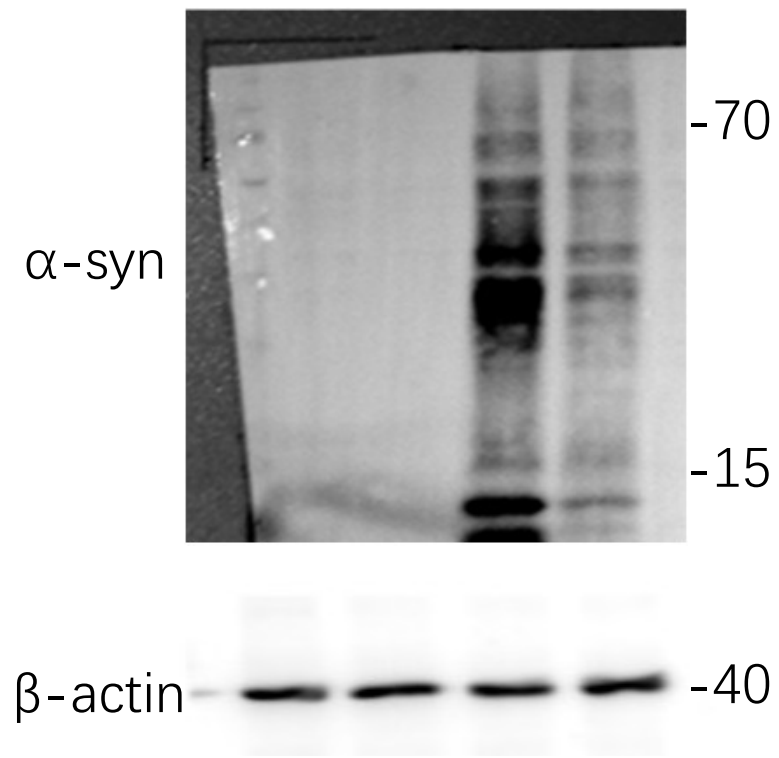

Figure 4 D

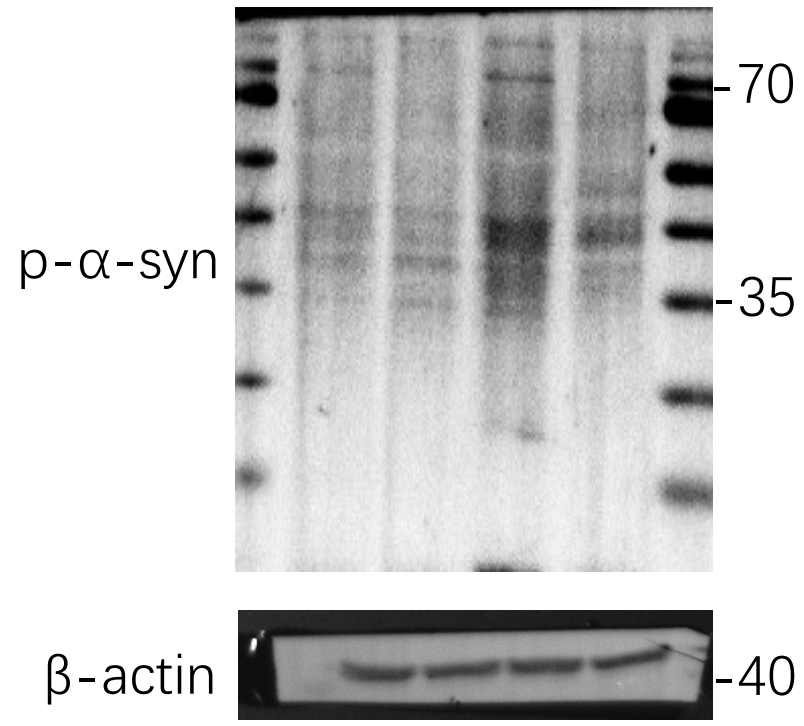

Figure 4 I

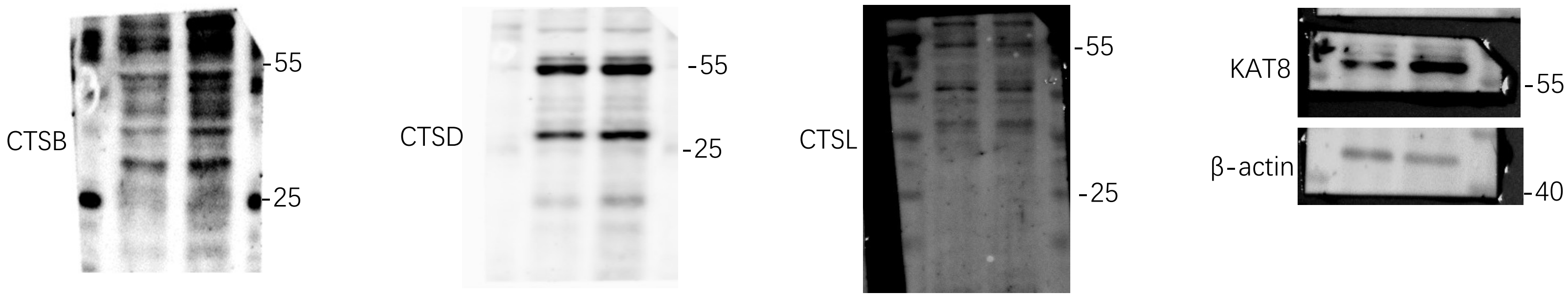

Figure 5 H

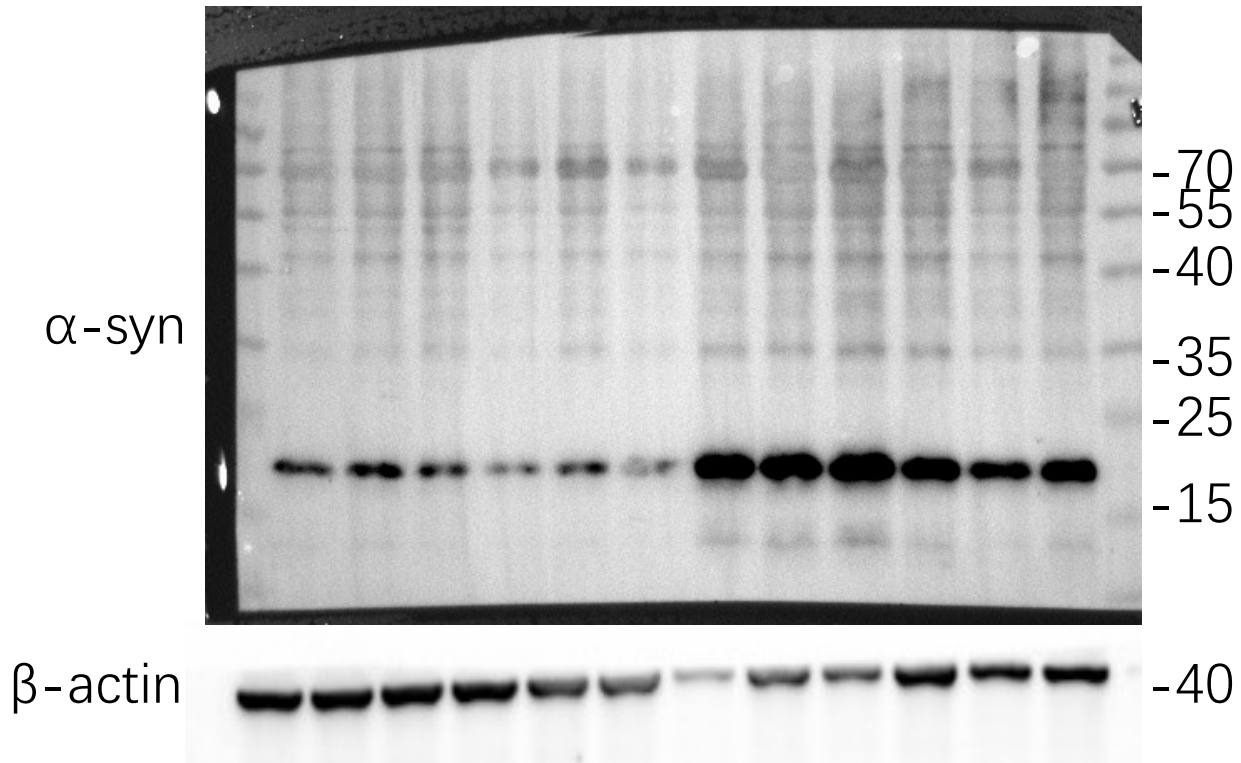

Figure 5 I

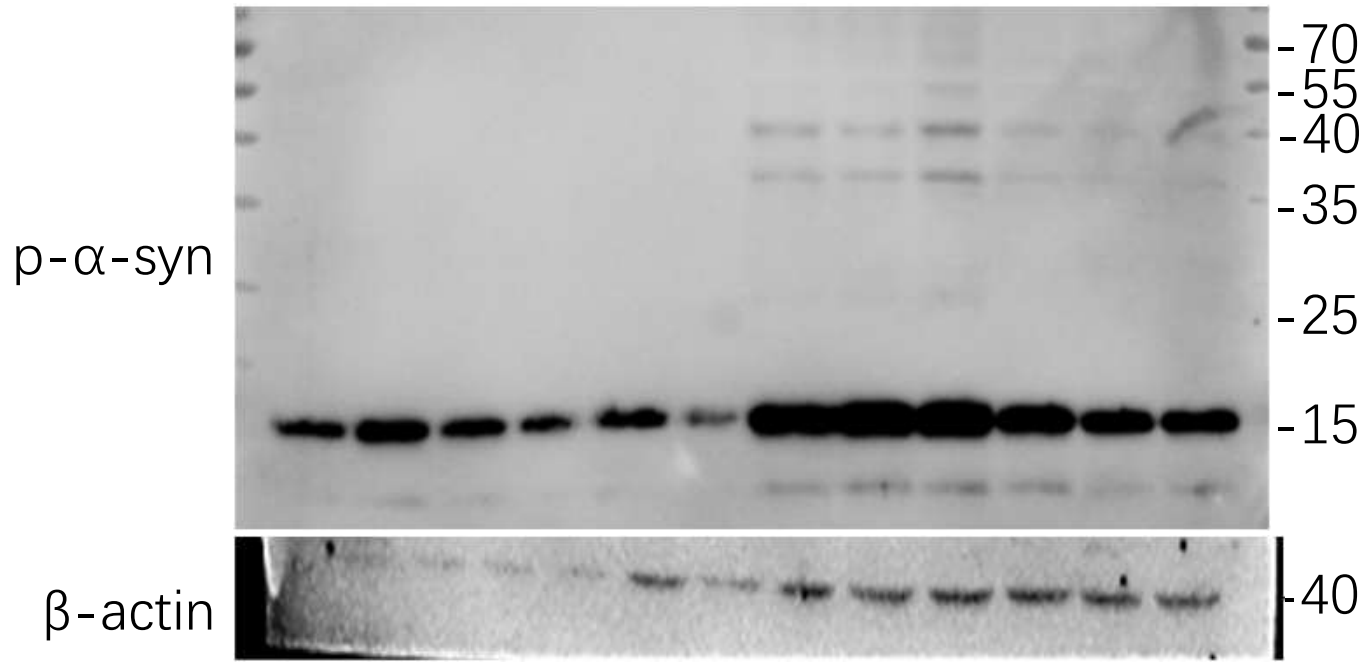

Figure S1 C

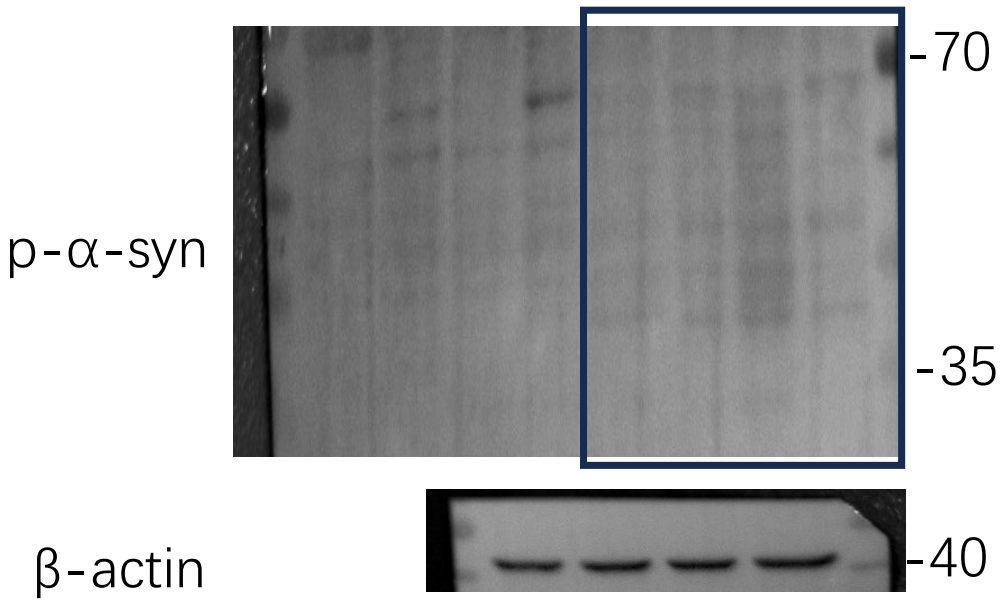

Figure S1 D

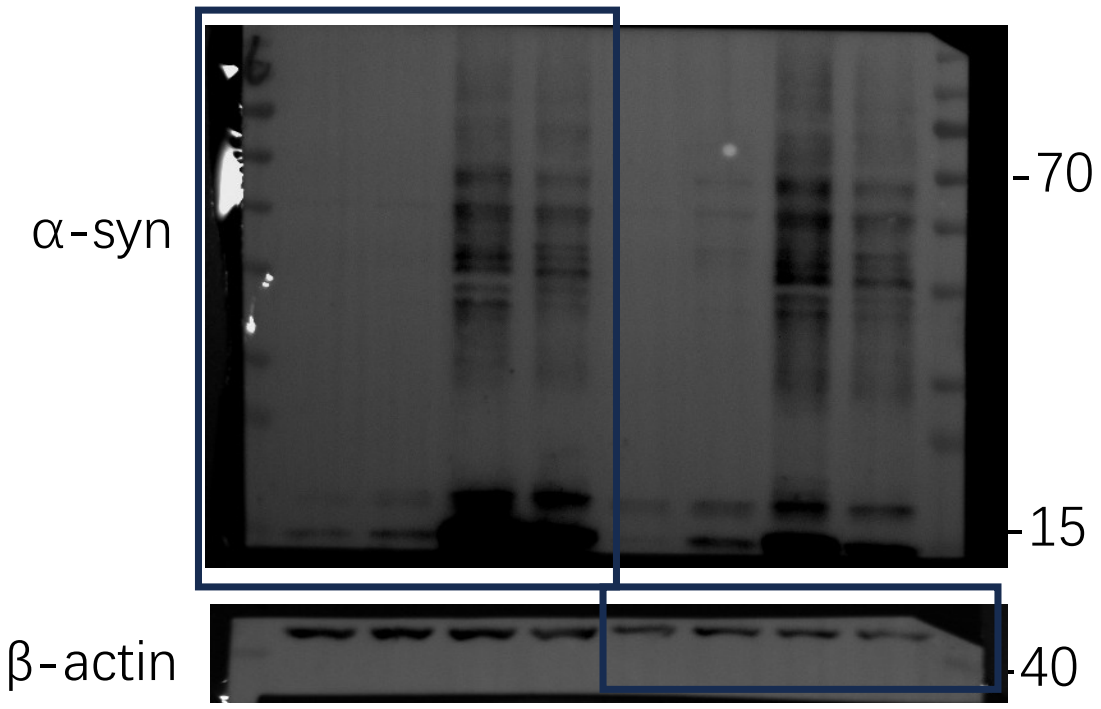

Figure S2 A

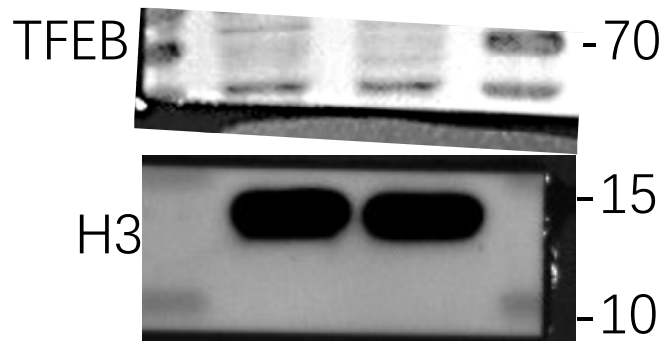

Figure S2 E

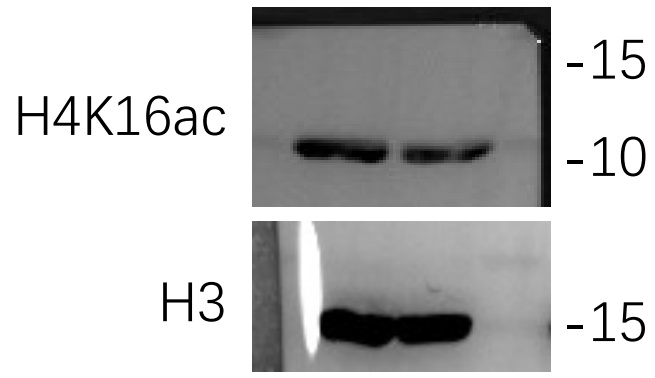

Figure S3 C

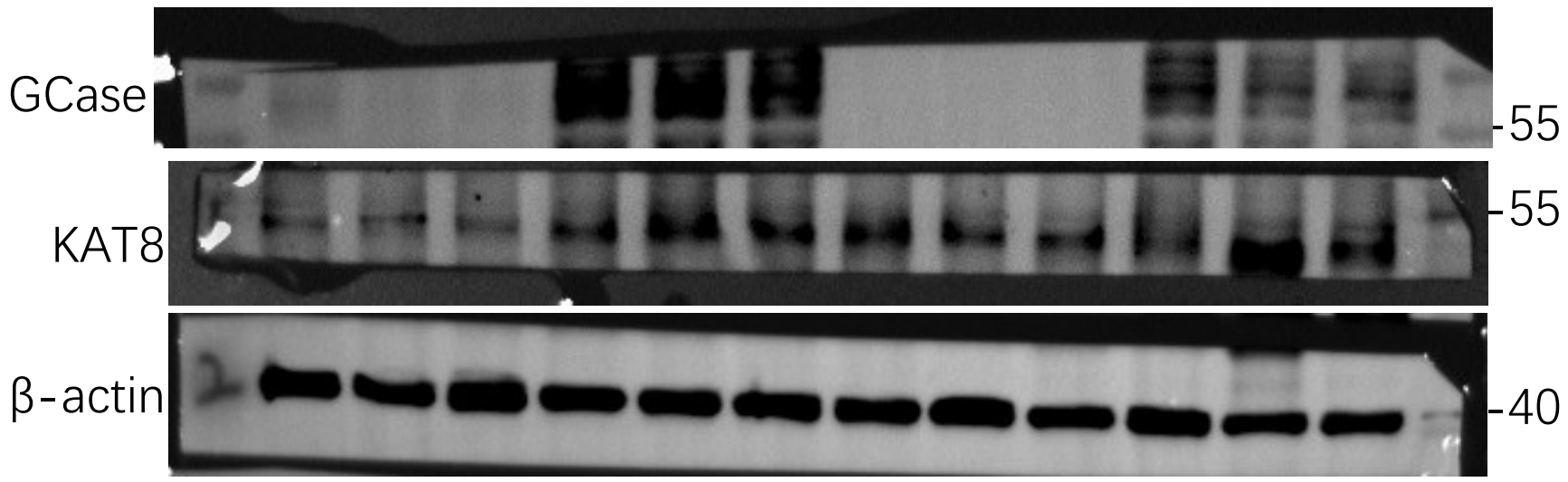

Figure S3 D

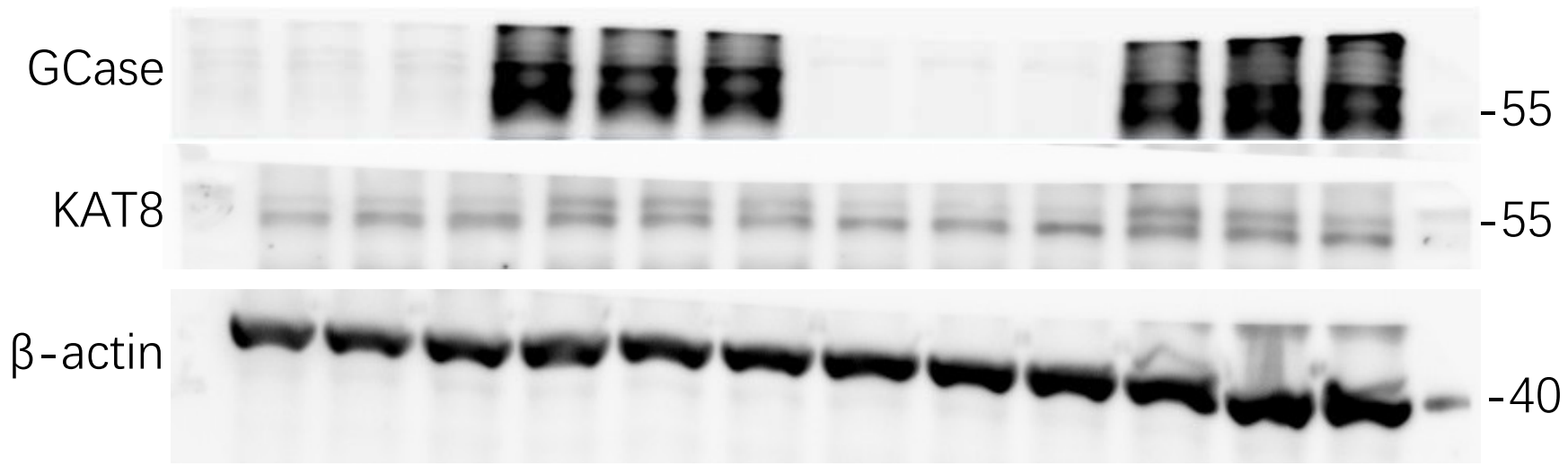

Figure S3 E

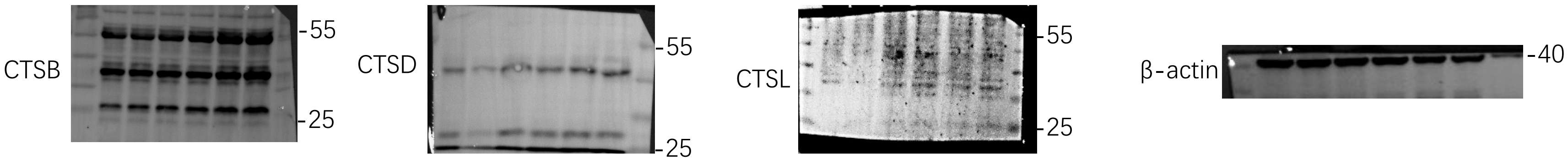

Figure S3 F

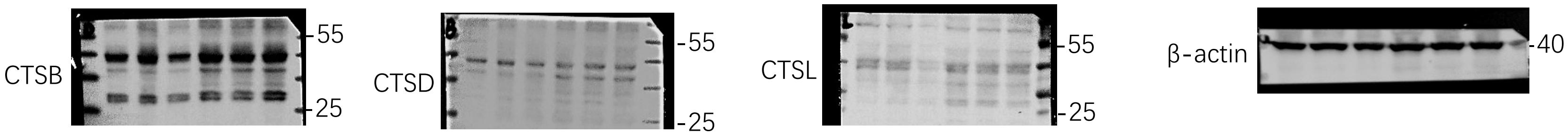

Figure1-B

PBS	1.28	0.89	0.75	1.06	0.95	PFF	11.21	14.94	13.66	6.82	9.09

Figure1-D

PBS	0.86	1.03	1.11			PFF	0.25	0.66	0.36		

Figure1-E

PBS	1.05	1.01	0.97	0.96		PFF	0.30	0.65	0.64	0.58	

Figure1-G

PBS	0.98	1.03	0.97	1.04	0.98	PFF	0.67	0.55	0.49	0.52	0.44

Figure2-A

OvCtr PBS	0.94	1.21	0.93	OvGBA1 PBS	1.42	1.58	1.53
OvCtrl PFF	0.73	0.68	0.66	OvGBA1 PFF	1.40	1.49	1.62

Figure2-C

OvCtr PBS	1.23	1.12	1.09	1.18	1.12	OvGBA1 PBS	1.12	1.11	1.09	1.24	1.15
OvCtrl PFF	11.32	12.32	8.60	10.98	10.78	OvGBA1 PFF	9.78	8.78	5.43	4.97	4.21

Figure2-E

OvCtr PBS	0.68	1.91	0.90	0.75	0.74	OvGBA1 PBS	1.15	1.18	1.79	1.64	1.18
OvCtrl PFF	0.14	0.31	0.46	0.60	0.46	OvGBA1 PFF	0.93	0.99	1.55	1.35	0.75

Figure2-G

DMSO				Leupeptin			
OvCtr PBS	OvGBA1 PBS	OvCtrl PFF	OvGBA1 PFF	OvCtr PBS	OvGBA1 PBS	OvCtrl PFF	OvGBA1 PFF
0.51	0.99	13.19	9.59	4.30	3.75	14.65	13.65
2.15	1.95	11.80	8.09	1.66	1.65	14.45	15.24
1.43	1.40	10.77	8.50	1.94	2.05	17.68	16.67

Figure2-H

	OvCtrl			OvGBA1		
Ctsb	0.94	1.04	1.02	1.31	1.36	1.37
Ctsd	0.90	1.09	1.02	1.53	1.62	1,66
Cstl	0.91	1.08	1.01	1.62	1.79	1.65

Figure2-J

	OvCtrl	OvGBA1		OvCtrl	OvGBA1		OvCtrl	OvGBA1		OvCtrl	OvGBA1		OvCtrl	OvGBA1
	0.93	1.15		1.10	1.99		1.19	3.12		0.97	3.41		1.14	1.93
**LAMP1**	1.02	1.19	**LAMP2**	0.97	1.81	**CTSB**	0.87	1.81	**CTSD**	1.19	6.14	**CTSL**	1.03	1.93
	1.05	1.26		0.53	2.46		1.10	2.54		0.89	2.95		1.32	2.16

Figure2-K

	OvCtrl	OvGBA1		OvCtrl	OvGBA1
	0.93	1.12		1.24	1.50
**CTSD/E**	1.04	1.19	**CTSB/L**	1.23	1.83
	0.94	1.05		0.72	1.69

Figure3-A

	Hat1	***Kat2a***	***Cbp***	***P300***	***Kat5***	***Kat6a***	***Kat6b***	***Kat7***	***Kat8***
	1.08	1.15	1.11	1.17	1.32	1.47	0.96	1.05	1.18
OvCtrl	1.03	0.86	0.95	1.08	0.75	0.79	0.98	0.94	0,94
	1.02	1.02	0.97	0.97	1.24	0.87	1.06	1.02	0.88
	1.46	1.17	1.28	1.00	0.75	1.13	1.15	1.28	1.66
OvGBA1	1.42	1.14	1.18	1.15	0.45	0.99	1.05	1.16	1.58
	1.14	1.00	1.27	1.20	0.89	1.07	1.03	1.16	1.54

Figure3-C

OvCtrl	0.83	1.03	1.14	OvGBA1	2.37	1.97	1.79

Figure3-E

OvCtrl	1.05	0.87	1.07	OvGBA1	1.48	1.47	1.34	OvGBA1+MC4033	1.04	0.77	1.10

Figure3-G

**Gcase/β-actin**	OvCtrl	0.82	0.79	0.81	MC4033	0.58	0.68	0.91
	OvGBA1	2.03	1.98	2.00	OvGBA1+MC4033	2.18	1.96	1.88
**CTSB/β-actin**	OvCtrl	1.03	0.81	1.24	MC4033	0.82	0.91	1.06
	OvGBA1	1.84	2.02	2.05	OvGBA1+MC4033	0.59	0.61	0.64
**CTSD/β-actin**	OvCtrl	0.81	1.03	1.24	MC4033	0.54	0.51	0.56
	OvGBA1	1.84	2.03	2.05	OvGBA1+MC4033	0.59	0.62	0.65
**CTSL/β-actin**	OvCtrl	1.00	1.04	1.01	MC4033	0.89	0.90	0.83
	OvGBA1	1.20	1.25	1.19	OvGBA1+MC4033	0.88	0.85	0.85

Figure3-H

			OvCtrl			OvGBA1			OvGBA1+MC4033	
LAMP1	IgG	0.84	1.28	0.93	0.90	0.75	1.48	2.50	0.39	0.83
	H4K16ac	2.60	2.12	2.00	21.70	20.26	20.30	3.43	4.78	3.68
CTSB	IgG	0.81	1.07	1.15	1.33	1.09	0.68	0.64	0.84	1.21
	H4K16ac	0.84	1.10	0.81	2.43	2.60	2.79	0.50	0.56	0.61
CTSD	IgG	1.05	0.93	1.04	1.58	0.85	0.74	0.75	1.35	0.97
	H4K16ac	1.33	1.37	1.43	2.33	2.79	2.02	0.93	0.45	0.75
CTSL	IgG	1.06	0.98	0.96	1.06	0.98	0.96	0.99	0.96	1.04
	H4K16ac	0.78	0.73	0.74	1.49	2.94	2.90	1.52	1.55	1.54

Figure3-J

DMSO				ACCS2-IN			
OvCtr PBS	OvGBA1 PBS	OvCtrl PFF	OvGBA1 PFF	OvCtr PBS	OvGBA1 PBS	OvCtrl PFF	OvGBA1 PFF
1.02	0.92	3.42	1.28	0.88	0.80	3.82	2.71
1.10	1.01	2.71	1.42	1.04	0.99	4.01	4.68
0.90	0.78	2.82	1.50	1.16	1.41	3.91	3.79

Figure4-B

DMSO				MC4033			
OvCtr PBS	OvGBA1 PBS	OvCtrl PFF	OvGBA1 PFF	OvCtr PBS	OvGBA1 PBS	OvCtrl PFF	OvGBA1 PFF
1.08	1.57	3.35	1.02	1.05	1.64	5.32	4.83
0.90	0.58	2.79	1.91	0.57	0.43	5.04	4.02
1.00	0.73	3.21	2.02	0.56	0.65	3.58	4.44

Figure4-E

OvCtr PBS	OvKAT8 PBS	OvCtrl PFF	OvKAT8 PFF
0.43	0.17	10.84	5.87
0.95	1.54	8.80	5.70
1.62	1.56	10.51	8.45

Figure4-F

OvCtr PBS	OvKAT8 PBS	OvCtrl PFF	OvKAT8 PFF
0.87	0.98	1.36	0.84
1.10	0.94	1.28	0.78
1.02	0.92	1.22	0.98

Figure4-H

OvCtr PBS	0.98	1.02	1.05	1.02	0.93	OvKAT8 PBS	1.06	0.98	0.90	1.04	1.02
OvCtrl PFF	5.41	5.31	4.95	5.25	6.93	OvGBA1 PFF	4.66	4.41	4.59	4.57	5.23

Figure4-J

	**CTSB/β-actin**			**CTSD/β-actin**			**CTSL/β-actin**		
OvCtrl	0.92	0.89	1.21	0.90	0.88	1.25	1.10	0.95	0.94
OvKAT8	4.23	3.18	3.54	2.81	2.20	3.13	1.80	2.40	3.16

Figure5-A

Front	OvCtrl+PBS	4.32	5.15	3.00	3.60	3.63	2.74	3.88	3.54	3.43	4.22
	OvGBA1+PBS	3.31	4.95	3.28	3.99	4.27	4.18	3.54	3.99	4.19	3.72
	OvCtrl+PFF	2.08	2.97	3.58	3.26	2.50	2.29	3.79	3.23	2.67	2.78
	OvGBA1+PFF	4.09	3.99	3.33	4.11	3.67	3.65	3.44	3.24	3.31	4.19
Hind	OvCtrl+PBS	4.63	5.41	5.24	4.59	4.70	4.76	6.57	6.82	6.32	6.56
	OvGBA1+PBS	6.43	6.84	7.08	5.78	4.65	7.42	5.29	5.05	6.29	6.03
	OvCtrl+PFF	5.04	4.27	4.70	4.78	4.32	4.52	3.61	5.77	4.71	5.26
	OvGBA1+PFF	6.39	5.87	5.79	6.18	5.20	5.35	7.41	5.72	4.43	5.96

Figure5-B

OvCtrl+PBS	1.07	1.05	0.91	0.98	1.00
OvGBA1+PBS	1.02	1.05	0.97	0.98	1.18
OvCtrl+PFF	0.83	0.85	0.87	0.87	0.85
OvGBA1+PFF	1.08	1.00	0.99	0.98	0.97

Figure5-C

OvCtrl+PBS	179	209	181	182	169
OvGBA1+PBS	168	147	200	186	181
OvCtrl+PFF	141	129	155	148	145
OvGBA1+PFF	201	163	183	182	198

Figure5-F

OvCtrl+PBS	1.00	1.00	0,92
OvGBA1+PBS	0.82	0.91	0.93
OvCtrl+PFF	1.72	1.66	1.68
OvGBA1+PFF	1.23	0.98	1.19

Figure5-G

OvCtrl+PBS	1.01	1.02	0,98
OvGBA1+PBS	0.80	0.91	0.92
OvCtrl+PFF	2.30	1.70	1.88
OvGBA1+PFF	0.98	1.32	1.22

Figure5-J

OvCtrl+PBS	1.04	1.47	0.48
OvGBA1+PBS	0.94	1.33	1.21
OvCtrl+PFF	5.57	4.77	3.54
OvGBA1+PFF	2.57	2.80	2.04

Figure5-K

OvCtrl+PBS	0.82	0.88	0.69
OvGBA1+PBS	0.53	0.68	0.31
OvCtrl+PFF	1.18	1.26	1.38
OvGBA1+PFF	0.76	0.76	0.84

Figure6

				Stritum						SNpc		
**KAT8**	EGFP	1.38	1.67	1.07	1.24	1.00	EGFP	1.02	1.11	1.03	0.92	0.90
	EGFP-GBA1	3.45	3.45	3.48	2.86	4.85	EGFP-GBA1	1.50	1.48	1.49	1.49	1.46
				Stritum			EGFP			SNpc		
**H4K16ac**	EGFP	1.14	0.94	0.95	0.91	1.07	EGFP-GBA1	0.92	0.81	0.93	1.28	1.22
	EGFP-GBA1	1.54	1.79	2.23	1.74	1.96	EGFP	1.71	2.21	1.85	1.36	1.35
				Stritum			EGFP-GBA1			SNpc		
**CTSB**	EGFP	0.08	0.66	1.09	1.85	1.36	EGFP	0.62	1.31	1.23	1.19	1.73
	EGFP-GBA1	2.52	2.70	3.70	1.38	1.54	EGFP-GBA1	1.57	3.14	2.60	3.26	3.64
				Stritum			EGFP			SNpc		
**CTSD**	EGFP	0.67	1.10	1.05	0.80	1.39	EGFP-GBA1	1.02	0.93	0.99	1.45	1.03
	EGFP-GBA1	1.87	1.86	2.59	1.73	1.78	EGFP	1.46	1.42	1.43	1.48	1.58
				Stritum			EGFP-GBA1			SNpc		
**CTSL**	EGFP	1.16	1.78	2.82	0.73	0.94	EGFP	0.97	1.07	1.16	0.95	1.03
	EGFP-GBA1	3.14	3.04	3.58	3.43	3.68	EGFP-GBA1	1.26	1.20	1.20	1.30	1.39

FigureS1-B

OvCtrl+PBS	1.05	0.97	1.01	0.90	1.05
OvGBA1+PBS	1.05	0.97	1.01	1.05	1.03
OvCtrl+PFF	2.70	3.60	2.76	2.46	2.40
OvGBA1+PFF	2.00	1.46	1.47	1.39	1.45

FigureS1-E

OvCtrl+PBS	1.00	1.00	1.18
OvGBA1+PBS	1.05	1.05	1.24
OvCtrl+PFF	3.53	3.53	3.40
OvGBA1+PFF	1.15	1.15	1.22

FigureS1-F

OvCtrl+PBS	1.07	1.40	0.52
OvGBA1+PBS	0.40	0.56	1.13
OvCtrl+PFF	15.00	19.90	17.12
OvGBA1+PFF	10.90	9.55	9.26

FigureS1-G

OvCtrl+PBS	0.95	1.04	1.00
OvGBA1+PBS	1.11	1.20	0.91
OvCtrl+PFF	1.72	1.65	1.62
OvGBA1+PFF	1.37	1.27	1.17

FigureS1-H

OvCtrl+PBS	0.94	1.08	1.00	1.06	0.90
OvGBA1+PBS	1.00	0.97	1.08	0.94	1.03
OvCtrl+PFF	0.55	0.46	0.49	0.50	0.46
OvGBA1+PFF	1.08	0.97	0.87	0.87	1.00

FigureS1-J

OvCtrl	1.14	0.50	0.99	0.99	1.37	OvGBA1	2.90	2.24	1.93	1.90	1.36

FigureS2-B

OvCtrl	0.89	0.98	1.18	OvGBA1	0.86	0.83	1.25

FigureS2-D

OvCtrl	14.12	10.11	11.23	12.20	14.10
OvGBA1	10.28	11.19	13.00	12.34	11.00
Torin1	80.00	79.14	65.98	92.00	91.34

FigureS2-F

DMSO	0.98	1.05	0.89	ACSS2-IN	0.54	0.64	0.74

FigureS2-G

CTSB					***CTSD***					***CTSL***				
OvCtrl DMSO	OVGBA1 DMSO	OVGBA1 MC4033	OVGBA1 NU 9056	OVGBA1 WM-3835	OvCtrl DMSO	OVGBA1 DMSO	OVGBA1 MC4033	OVGBA1 NU 9056	OVGBA1 WM-3835	OvCtrl DMSO	OVGBA1 DMSO	OVGBA1 MC4033	OVGBA1 NU 9056	OVGBA1 WM-3835
0.96	1.95	0.72	0.96	2.04	1.00	2.48	0.55	1.37	1.65	0.97	3.00	1.05	2.27	2.78
1.01	2.31	0.98	1.59	2.06	0.90	2.48	0.70	1.77	1.44	0.99	2.11	1.30	2.41	2.63
1.02	2.10	0.85	1.71	1.82	1.12	2.39	0.72	1.33	1.28	1.04	1.62	1.32	3.25	2.34

FigureS2-J

OvCtrl DMSO	OVGBA1 DMSO	OVGBA1 MC4033	OVGBA1 NU 9056	OVGBA1 WM-3835
1.08	2.04	1.22	2.19	1.88
0.95	2.27	1.22	2.02	1.88
0.81	2.17	1.35	1.82	2.02
1.08	2.05	1.22	2.04	1.88
1.08	2.04	1.22	2.09	2.09

FigureS2-K

OvCtrl DMSO	OVGBA1 DMSO	OVGBA1 MC4033	OVGBA1 NU 9056	OVGBA1 WM-3835
1.13	2.97	1.11	1.95	3.25
1.04	2.79	1.22	2.97	2.82
0.99	3.02	1.57	2.83	2.79
0.95	2.76	2.12	2.86	2.67
0.90	3.16	2.01	2.93	2.70

FigureS2-L

DMSO	1.01	1.02	0.98	CBE	0.79	0.50	0.50

FigureS2-M

	KAT8			CTSB			CTSD			CTSL			LAMP1		
OvCtrl	1.05	1.09	0.85	0.90	0.96	1.15	0.75	1.12	1.18	0.69	1.15	1.26	0.88	1.20	0.88
OVGBA1-N40SP	0.75	1.01	0.88	0.67	1.23	1.10	0.54	1.03	0.72	0.42	0.87	0.66	0.50	0.97	0.92
OVGBA1-L444P	1.18	1.11	1.03	1.09	0.74	0.85	1.27	1.07	0.91	1.12	1.07	0.87	1.29	0.97	1.18

FigureS2-O

OvCtrl	1.18	1.12	1.11	1.03	1.23
OVGBA1-N370S	0.85	0.95	0.86	0.95	1.13
OVGBA1-L444P	1.23	1.07	1.00	0.94	1.03

FigureS3-A

EGFP	1.64	1..87	0.42	0.45	0.65
EGFP-GBA1	5.70	4.40	2.80	4.00	3.90

FigureS3-B

EGFP	1.33	0.87	0.42	0.94	0.96
EGFP-GBA1	2.50	4.00	4.80	4.42	3.74

FigureS3-C

**KAT8/β-actin**	EGFP PBS	EGFP-GBA1 PBS	EGFP PFF	EGFP-GBA1 PFF
	0.79	1.47	1.41	1.51
	1.07	1.78	1.46	1.46
	1.15	1.39	1.37	1.57

**GCase/β-actin**	EGFP PBS	EGFP-GBA1 PBS	EGFP PFF	EGFP-GBA1 PFF
	0.90	21.71	0.89	14.60
	1.32	19.39	0.65	15.49
	0.78	19.35	0.33	13.64

FigureS3-D

**KAT8/β-actin**	EGFP PBS	EGFP-GBA1 PBS	EGFP PFF	EGFP-GBA1 PFF
	0.94	1.43	1.52	2.19
	1.20	1.81	1.22	1.74
	0.86	1.47	0.15	1.55

**GCase/β-actin**	EGFP PBS	EGFP-GBA1 PBS	EGFP PFF	EGFP-GBA1 PFF
	1.28	7.13	0.16	4.48
	0.79	6.76	1.10	2.97
	0.93	6.37	0.18	3.68

FigureS3-G

	**CTSB/β-actin**				**CTSD/β-actin**				**CTSL/β-actin**	
EGFP	0.93	0.90	1.03	EGFP	1.18	0.72	1.10	EGFP	0.83	0.60	1.45
EGFP-GBA1	1.17	1.41	1.26	EGFP-GBA1	1.36	1.39	1.39	EGFP-GBA1	1.66	1.87	1.45

FigureS3-H

	**CTSB/β-actin**				**CTSD/β-actin**				**CTSL/β-actin**	
EGFP	0.74	1.09	1.15	EGFP	0.95	1.06	0.99	EGFP	0.90	1.04	1.07
EGFP-GBA1	1.51	1.39	1.29	EGFP-GBA1	1.37	1.70	1.69	EGFP-GBA1	1.20	1.24	1.52
