# Supplementary material for: Lysine acetyltransferase 8-mediated histone acetylation, regulated by GBA1, is associated with lysosomal function related to α-Synuclein pathology
Source: Cell Death Dis. 2026 Jun 8;17(1):711. doi: 10.1038/s41419-026-08928-2 (PMC13462883; doi:10.1038/s41419-026-08928-2)
Supplement: Supplementary file 2 [file 41419_2026_8928_MOESM1_ESM.docx]

**Supplementary Text**

**Lysine Acetyltransferase 8-Mediated Histone Acetylation Regulated by GBA1 is Associated with Lysosomal Function Related to α-Synuclein Pathology**

Yifan Cao^1^, Zhixiong Zhang^1^, Xinbei Gu^1^, Haifeng Lu^2^, Jin Wu^1^, Chuang Zhang^1^, Xinyue Huang^1^, Haitao Qin^3^, Feng Liu^3^, Zhenhua Liu^4^, Beisha Tang^4^, Xiaojun Lu^5^, Haigang Ren^1^, Hongyang Sun^1*^, Rui Wang^1*^, Guanghui Wang^1,6,7*^

Figs. S1 to S3

**GBA1 restores lysosomal function by promoting lysosomal biogenesis**

Immunocytochemical staining also revealed that *GBA1* overexpression mitigated the PFF-induced pathology of α-syn, which was labeled with an antibody against S129-phosphorylated α-syn (Fig. S1, A and B). Similar results were obtained via Western blot analysis (Fig. S1, C to F). We further validated the neuroprotective effect of *GBA1* overexpression on α-syn PFFs. Lactate dehydrogenase (LDH) release assays revealed that *GBA1* overexpression inhibited PFF-induced neurotoxicity (Fig. S1, G). Immunocytochemical staining with an antibody against NeuN, a specific neuronal marker, revealed that *GBA1* overexpression inhibited PFF-induced neuronal loss (Fig. S1, H). Moreover, the activities of cathepsins and lysosomal proteases also increased (Fig. S1, I and J).

**GBA1 increases lysosomal function through the histone acetyltransferase KAT8**

To determine whether *GBA1* overexpression enhances lysosomal function through TFEB-mediated biosynthesis, we analyzed TFEB expression in the nucleus via Western blotting. In primary neurons, *GBA1* overexpression did not affect intranuclear TFEB expression in cells (Fig. S2, A and B). Additionally, we transfected U2OS cells with Flag and Flag-*GBA1* plasmids and labeled them with endogenous TFEB via immunofluorescence. As a positive control for monitoring TFEB nuclear translocation, the mTOR inhibitor Torin 1 was used. In U2OS cells treated with Torin 1, TFEB nuclear translocation was observed (Fig. S2, C and D). However, nuclear translocation of TFEB was not observed in *GBA1*-overexpressing cells (Fig. S2, C and D), indicating that TFEB does not mediate the upregulation of lysosome-associated gene expression in *GBA1*-overexpressing cells. To further identify the effects of H4K16 modification on α-syn aggregation, we treated primary neurons with the ACSS2 inhibitor ACSS2-IN for 72 hours (h). Western blot analysis revealed a reduction in the nuclear level of H4K16ac, confirming that ACSS2 activity is crucial for maintaining H4K16ac acetylation, and with the inhibition of ACSS2, H4K16ac modification was decreased (Fig. S2, E and F). Given that KAT5 and KAT7 are also regulated by GBA1, we investigated whether they contribute to GBA1-mediated lysosomal regulation. In GBA-overexpressing U2OS cells inhibition of KAT5 and KAT7 modestly reduced the transcription of a limited subset of lysosomal proteins, but the effects were weaker and narrower in scope compared with KAT8 (Fig. S2, G). Meanwhile, U2OS cells in which *GBA1* were overexpressed were treated with KAT5, KAT7, and KAT8 inhibitors to examine whether these three acetyltransferases mediate the *GBA1*‑regulated lysosomal function. Representative images of DQ‑BSA assays (S2, H) and LysoTracker staining (S2, I), along with quantitative analyses of the fluorescence intensities for DQ‑BSA (S2, J) and LysoTracker staining (S2, K), revealed that only the KAT8 inhibitor attenuated the *GBA1*‑mediated increase in lysosomal hydrolytic activity (S2, H, J) and in lysosomal acidification (S2, I, K). Together, these findings indicate that KAT8 acts as a specific regulator among the KAT family members in response to *GBA1*. To determine whether the regulation of KAT8 by *GBA1* depends on GCase enzymatic activity, primary neurons were treated with a GCase inhibitor CBE, and *Kat8* mRNA levels were measured (S2, L). The results demonstrated that decreased GCase activity markedly downregulates *Kat8* expression. Overexpression of pathological *GBA1* mutants (*N370S* and *L444P*) in U2OS cells failed to increase the transcript levels of KAT8 or lysosome‑related genes (S2, M), and enhanced lysosomal proteolytic activity (S2, N, O).

**GBA1 attenuates α-syn pathology in an α-syn PFF mouse model**

Given that *GBA1* overexpression enhances lysosomal biogenesis and reduces α-syn aggregation *in vitro*, we next determined whether *GBA1* overexpression could mitigate α-syn pathology *in vivo* in an α-syn PFF mouse model. Using an AAV-mediated gene delivery strategy with AAV-EGFP-*GBA1* (or AAV-EGFP as a control), widespread expression of *GBA1* was observed four months after the AAVs were injected into the striatum in adult mice (Fig. S3, A to D). We then examined the effects of *GBA1* overexpression on KAT8 expression and lysosomal biogenesis. *GBA1* overexpression resulted in increased levels of KAT8 in the striatum and midbrain (Fig. 6, A and B and Fig. S3, C and D). Moreover, in the mice in which *GBA1* was overexpressed, increases in the expression of the lysosomal cathepsins CTSB, CTSD, and CTSL were observed in the striatum and midbrain, as indicated by Western blotting (Fig. S3, E to H).

Fig. S1 to S3

**Fig. S1** **Overexpression of GBA1 rescues α-syn aggregation caused by α-syn PFF treatment and** **enhances lysosomal hydrolase activity in primary neurons**

**A** to **H**, Primary neurons at 6 DIV were transduced with LV containing Flag-*GBA1* or Flag. PBS or 1 µg/mL PFFs were added to the neurons at 7 DIV for 14 days, after which they were processed for analysis. Representative images of immunofluorescence staining for p-α-syn and Map2 (**A**). Scale bar, 10 μm. Quantification of p-α-syn intensity (**B**), (***p* < 0.01, ****p* < 0.001, n = 5/group). Neuron lysates were sequentially extracted in 1% TX-100 followed by 2% SDS (TX-insoluble). Immunoblots of neuron lysates containing misfolded α-syn (**C**) and p-α-syn (**D**) are shown. β-actin served as a loading control. The protein levels of misfolded α-syn (**E**) and p-α-syn (**F**) were normalized to those of β-actin (***p* < 0.01, *****p* < 0.0001, n = 5/group). Lactate dehydrogenase (LDH) assay analysis of primary neurons **(G)** (**p* < 0.05, n = 3/group). Quantification of the number of Neuron N **(H)** (*****p* < 0.0001, n = 5/group). DQ-BSA was added to the cells at a final concentration of 50 µg/ml in prewarmed medium for 8 h. Representative immunofluorescence images after staining with DQ-BSA (**I**). Scale bar, 10 μm. Quantiﬁcation of the DQ-BSA intensity (**J**). (***p* < 0.01, n = 5/group). The data are presented as the means ± SEMs. Statistical significance was determined by two-way ANOVA in (**B**) and (**E** to **H**) or an unpaired *t* test in (**J**).

**Fig. S2 GBA1 influences biosynthesis independently of TFEB.**

**A** and **B**, Primary neurons at 6 DIV were transduced with LV containing Flag-*GBA1* or Flag for 5 days. The protein levels of TFEB were visualized by immunoblotting (**A**). Quantiﬁcation of the indicated protein levels normalized to H3 (**B**). (ns indicates not significant, n = 3/group). **C** to **F**, U2OS cells were transfected with a vector encoding Flag-*GBA1* and Flag for 48 h. Then, Torin-1 (100 nM) or DMSO was used to treat the cells for 8 h. Representative images of immunofluorescence staining for TFEB and DAPI (**C**); scale bar, 10 µm. The percentage of TFEB that was localized to the nucleus (**D**). (*****p* < 0.0001, mean ± SD, ns indicates not significant, n > 50 cells, five fluorescent fields were selected for counting from three independent experiments). **E** and **F**, Primary neurons were pretreated with ACSS2-IN (2.5 μM) at 5 DIV to inhibit histone acetylation for 72 h and then transduced with LVs containing EGFP-*GBA1* or EGFP at 6 DIV for 5 days. The H4K16ac levels were visualized by immunoblotting (**E**), and the quantified protein levels were normalized to those of H3 (**F**). (**p* < 0.05, n = 3/group). **G to K**, U2OS cells were transfected with a vector encoding Flag-*GBA1* and Flag for 48 h. Then, MC4033 (20 μM), NU 9056 (20 μM) and WM-3835 (20 μM) were used to treat the cells for 18 h. The qPCR analysis of the mRNA levels of lysosomal-related genes (*CTSB, CTSD,* and *CTSL*) were in (**G**), (***p* < 0.01, *****p* < 0.0001, n = 3/group). DQ-BSA was added to the cells at a final concentration of 50 µg/ml in prewarmed medium for 8 h. The images after DQ-BSA treatment (**H**) or LysoTracker staining (**I**) were shown. Scale bar, 20 μm. Quantiﬁcation of the DQ-BSA intensity (**J**) or the LysoTracker intensity (**K**) were shown. (*****p* <0.0001, n = 5/group). Scale bars, 20 μm. **L**, Primary neurons at 6 DIV were treated with CBE for 24 h. The qPCR analyses of the mRNA levels of *Kat8* were shown (**p* < 0.1, n = 3/group). **M** to **O**, U2OS cells were transfected with plasmids encoding Flag alone (OvCtrl), Flag-*GBA1^N370S^* (OvGBA1*^N370S^*), or Flag-*GBA1^L444P^* (OvGBA1*^L444P^*) for 48 h. The qPCR analysis of the mRNA levels of *KAT8, CTSB, CTSD*, *CTSL* and *LAMP1* were shown (**M**), Representative images after staining with DQ-BSA were shown (**N**). Scale bar, 20 μm. Quantiﬁcation of the DQ-BSA intensity were shown (**O**) (n = 5/group). The data are presented as the means ± SEMs. Statistical significance was determined by an unpaired *t* test in (**B**), (**D**), (**F**), (**G**), (**J** to **L**), (**M**) and (**O**).

**Fig. S3** **Overexpression of GBA1 led to elevated levels of KAT8 and cathepsin family proteins in the mouse model.**

The mice received injections of AAV-EGFP-*GBA1* (2E10 vg/mouse) or AAV-EGFP (2E10 vg/mouse) in the bilateral dorsal striatum followed by 2.5 µg/side PFFs or PBS 1 month later. Behavioral testing and histological analysis were performed 3 months after the injection of PFFs. **A** and **B**, Representative images of staining for GCase in the striatum (**A**) and SNpc (**B**). Scale bar, 25 μm. The quantification of the percentage of the area covered by GCase staining in the mice is shown on the right. (*****p* < 0.0001, n = 5/group). **C** and **D**, Western blot analysis of GCase and KAT8 protein levels in the striatum (**C**) and midbrain (**D**). Quantiﬁcation of the indicated protein levels normalized to that of β-actin is shown on the right. (**p* < 0.05, ***p* < 0.01, *****p* < 0.0001, n = 3/group). **E** and **F**, Western blot analysis of CTSB, CTSD and CTSL protein levels in the striatum (**E**) and midbrain (**F**). Quantiﬁcation of the indicated protein levels normalized to that of β-actin is shown in (**G** and **H**) (**p* < 0.05, ***p* < 0.01, n = 3/group). The data are presented as the means ± SEMs. Statistical significance was determined by an unpaired *t* test in (**A**), (**B**), (**G**) and (**H**) or two-way ANOVA in (**C**) and (**D**).
