# Supplementary material for: Lysine acetyltransferase 8-mediated histone acetylation, regulated by GBA1, is associated with lysosomal function related to α-Synuclein pathology
Source: Cell Death Dis. 2026 Jun 8;17(1):711. doi: 10.1038/s41419-026-08928-2 (PMC13462883; doi:10.1038/s41419-026-08928-2)
Supplement: Supplementary file 3 [file 41419_2026_8928_MOESM6_ESM.docx]

Supplementary table: Primers for qPCR

| Gene | Species | Primer | Sequence |
| --- | --- | --- | --- |
| *Ctsb* | Mouse | Forward | 5'-TCCTTGATCCTTCTTTCTTGCC-3' |
|  |  | Reverse | 5'-ACAGTGCCACACAGCTTCTTC-3' |
| *Ctsd* | Mouse | Forward | 5'-GCTTCCGGTCTTTGACAACCT-3' |
|  |  | Reverse | 5'-CACCAAGCATTAGTTCTCCTCC-3' |
| *Ctsl* | Mouse | Forward | 5'-ATCAAACCTTTAGTGCAGAGTGG-3' |
|  |  | Reverse | 5'-CTGTATTCCCCGTTGTGTAGC-3' |
| *Actin* | Mouse | Forward | 5'-GACCTGACTGACTACCTC-3' |
|  |  | Reverse | 5'-GACAGCGAGGCCAGGATG-3' |
| *Hat1* | Mouse | Forward | 5'-AAGTGTAACACCAACACAGCA-3' |
|  |  | Reverse | 5'-CGAAAGCAGTTTCATCATCCCC-3' |
| *Kat2a* | Mouse | Forward | 5'-AAGGCCAATGAAACCTGCAAG-3' |
|  |  | Reverse | 5'-CTCACAGCTACGGCACAACTC-3' |
| *Cbp* | Mouse | Forward | 5'-GGCTTCTCCGCGAATGACAA-3' |
|  |  | Reverse | 5'-GTTTGGACGCAGCATCTGGA-3' |
| *P300* | Mouse | Forward | 5'-TTCAGCCAAGCGGCCTAAA-3' |
|  |  | Reverse | 5'-CGCCACCATTGGTTAGTCCC-3' |
| *Kat5* | Mouse | Forward | 5'-TCCCGGTCCAGATCACACTC-3' |
|  |  | Reverse | 5'-ACCTTCCGTTTCGTTGAGCG-3' |
| *Kat6a* | Mouse | Forward | 5'-ATGGTAAAACTCGCTAACCCG-3' |
|  |  | Reverse | 5'-CGTCCCGTCTTTGACGCTC-3' |
| *Kat7* | Mouse | Forward | 5'-ATGCCGCGAAGGAAGAGAAAT-3' |
|  |  | Reverse | 5'-TCTTGGGAACTCTGGCTTAGC-3' |
| *Kat8* | Mouse | Forward | 5'-ACGAGGCGATCACCAAAGTG-3' |
|  |  | Reverse | 5'-AAGCGGTAGCTCTTCTCGAAC-3' |
| *CTSB* | Human | Forward | 5'-GAGCTGGTCAACTATGTCAACA-3' |
|  |  | Reverse | 5'-GCTCATGTCCACGTTGTAGAAGT-3' |
| *CTSD* | Human | Forward | 5'-TGCTCAAGAACTACATGGACGC-3' |
|  |  | Reverse | 5'-CGAAGACGACTGTGAAGCACT-3' |
| *CTSL* | Human | Forward | 5'-CTTTTGCCTGGGAATTGCCTC -3' |
|  |  | Reverse | 5'-CATCGCCTTCCACTTGGTC-3' |
| *LAMP1* | Human | Forward | 5'-TCTCAGTGAACTACGACACCA-3' |
|  |  | Reverse | 5'-AGTGTATGTCCTCTTCCAAAAGC-3' |
| *ACTIN* | Human | Forward | 5-CATGTACGTTGCTATCCAGGC-3' |
|  |  | Reverse | 5'-CTCCTTAATGTCACGCACGAT-3' |
